# Activity Profiling of Nitro‐Substituted Di(Hetero)Aryl 1,3,4‐ and 1,2,4‐Oxadiazoles: Antimicrobial, Cholinesterase Inhibition and Antioxidant Potential

**DOI:** 10.1002/ardp.70188

**Published:** 2026-01-20

**Authors:** Enikő Šikorová, Šárka Štěpánková, Eva Frýbová, Klára Konečná, Jana Korbielová, Markéta Švarcová, Ondřej Janďourek, Václav Pflégr, Szilvia Bősze, Martin Krátký

**Affiliations:** ^1^ Department of Organic and Bioorganic Chemistry, Faculty of Pharmacy in Hradec Králové Charles University Hradec Králové Czech Republic; ^2^ Department of Biological and Biochemical Sciences, Faculty of Chemical Technology University of Pardubice Pardubice Czech Republic; ^3^ Department of Biological and Medical Sciences, Faculty of Pharmacy in Hradec Králové Charles University Hradec Králové Czech Republic; ^4^ Laboratory for Mycobacterial Diagnostics and Tuberculosis Regional Institute of Public Health in Ostrava Ostrava Czech Republic; ^5^ HUN‐REN–ELTE Research Group of Peptide Chemistry, Hungarian Research Network, Institute of Chemistry ELTE Eötvös Loránd University Budapest Hungary; ^6^ Department of Genetics, Cell‐ and Immunobiology, Faculty of Medicine Semmelweis University Budapest Hungary

**Keywords:** antimicrobial activity, antioxidant activity, cholinesterases, nitro compounds, oxadiazoles

## Abstract

Oxadiazole derivatives represent a promising scaffold for drug discovery, offering therapeutic potential, notably against neurodegenerative diseases and microbial infections. Based on molecular hybridisation approach utilising bioactive oxadiazoles, pyridine‐, quinoline‐ and nitrophenyl‐based compounds, we designed novel 1,3,4‐ and 1,2,4‐oxadiazole derivatives containing nitro group(s), evaluated their enzyme inhibition, antimicrobial, and antioxidant properties, and analysed their structure–activity relationships (SAR). Comprehensive activity assessments of them and their synthetic precursors revealed robust inhibitory activity against acetylcholinesterase with IC_50_ values as low as 1.47 µM and butyrylcholinesterase (IC_50_ ≥ 45.09 μM), outperforming rivastigmine in several cases. Mechanistic insights *via* molecular docking unveiled unique binding modes for cholinesterases inhibition. Antimicrobial screening demonstrated potent activity (MIC ≥ 2 μM) of several compounds against *Mycobacterium tuberculosis*, atypical mycobacteria, Gram‐positive bacteria including methicillin‐resistant *Staphylococcus aureus*, and mould *Trichophyton interdigitale*. The antioxidant evaluation identified derivatives' free‐radical scavenging potential. SAR analysis identified essential structural features, favouring 3,5‐dinitrophenyl moiety and 1,3,4‐oxadiazoles over 1,2,4‐isomers and 1,2‐diacylhydrazine precursors. In summary, novel candidates for addressing challenges in treating infectious diseases and disorders related to insufficient acetylcholine transmission were identified.

## Graphical abstract

Novel nitro‐substituted diaryl 1,3,4‐ and 1,2,4‐oxadiazoles were synthesised via molecular hybridisation of bioactive heterocycles. Selected derivatives exhibited potent antimicrobial activity, including against *Mycobacterium tuberculosis* and *Staphylococcus aureus*, and cholinesterase inhibitory activity, with promising selectivity. Structure–activity relationship analysis highlighted key structural motifs for antimicrobial and neuroprotective potential.

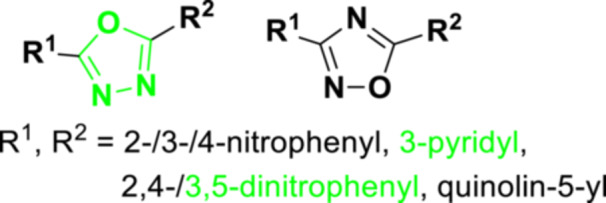

## 1 Introduction

**Figure 1:**
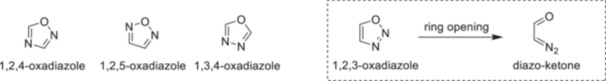
Three stable oxadiazole regioisomers and the unstable 1,2,3‐oxadiazole.

Heterocycles have become increasingly important scaffolds in medicinal chemistry and drug development, with more than 85% of bioactive compounds featuring at least one heterocycle. The most prevalent are five‐ and six‐membered rings, particularly those that incorporate nitrogen, alone or in combinations with other heteroatoms, such as oxygen and/or sulphur [1, 2, 3]. Within this group, oxadiazoles – five‐membered rings containing one oxygen and two nitrogen atoms – have attracted significant attention. There are three stable regioisomers of unsubstituted oxadiazole: 1,2,4‐, 1,2,5‐, and 1,3,4‐isomers, while the 1,2,3‐oxadiazole is highly unstable and prone to ring opening due to the weakness of its N‐O sigma bond, forming the corresponding diazo‐ketone, which is energetically more favourable by nearly 9 kcal/mol (Figure 1) [4, 5, 6].

Among these isomers, 1,2,4‐ and particularly 1,3,4‐oxadiazoles have been extensively explored in drug development [7] due to diverse biological activities–ranging from analgesic, anti‐inflammatory, antibacterial, antimycobacterial, antifungal, antiparasitic, antiviral, anticancer and antidiabetic, which have been summarised in numerous reviews [1, 4, 8, 9, 10, 11, 12, 13]. In many cases, the oxadiazole ring is part of the pharmacophore, contributing to ligand binding at the target's active site [1, 14]. Moreover, oxadiazoles often enhance the physicochemical properties of drug candidates and serve as effective bioisosters for various carbonyl‐based functional groups such as esters, amides, hydrazides, carbamates, or even benzene [7].

Oxadiazoles can be further functionalized at two positions with various scaffolds, offering opportunities to enhance their pharmacodynamic and pharmacokinetic properties. One prominent strategy involves substitution with additional heterocycles such as pyridine and quinoline, which are themselves pharmacologically privileged scaffolds. These heteroaryl motifs are found in numerous approved drugs including the antituberculotics isoniazid (INH) and bedaquiline, as well as antimalarials primaquine and chloroquine [15, 16, 17]. The introduction of a nitro group is another effective approach, although this strategy is often avoided in drug development due to concerns about genotoxicity and mutagenicity [18]. Nevertheless, nitro group remain integral pharmacophore in many approved drugs (e.g., the antibacterial furamizole), and recent approvals further support its therapeutic relevance. Notably, delamanid and pretomanid, two nitroimidazole‐based drugs used to treat drug‐resistant tuberculosis, function as prodrugs with a nitro group‐dependent mechanism of action that is selective in mycobacteria, thus minimalizing genotoxic risk [19].

**Figure 2:**
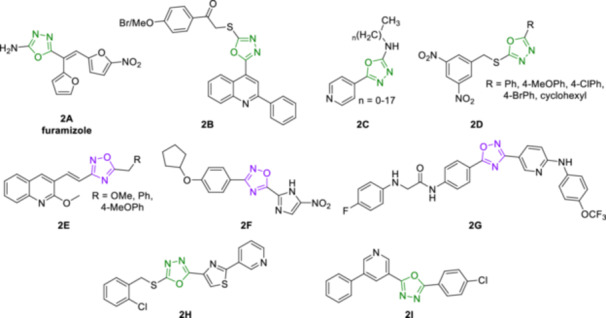
1,3,4‐oxadiazoles and 1,2,4‐oxadiazoles with antimicrobial (**2A**–**2F**) and potential anti‐neurodegenerative action (**2G**–**2I**).

The therapeutic value of oxadiazoles is illustrated by various literature reports describing their use in bioactive scaffolds. For example, the 1,3,4‐oxadiazole ring together with an aromatic nitro group can be found in the approved antibacterial agent furamizole [9] (**2A**, Figure 2). Several hybrid molecules have also been designed to enhance antimicrobial activity. For example, quinoline‐1,3,4‐oxadiazole hybrids [20] **2B** have demonstrated potent antibacterial activity through dual inhibition of bacterial DNA gyrase and topoisomerase IV (Figure 2). Similarly, INH‐1,3,4‐oxadiazole analogues [21] **2C** exhibited antimycobacterial effects with mechanism of action distinct from the parent compound, maintaining efficacy even against resistant strains (Figure 2). Importantly, nitro‐containing 1,3,4‐oxadiazoles [22] **2D** have displayed high and selective antimycobacterial activity, where the nitro moiety was proven essential for the efficacy (Figure 2). In the case of 1,2,4‐oxadiazoles (Figure 2), their quinoline‐hybrids [23] **2E** showed submicromolar minimum inhibitory concentration (MIC) values, as well as the nitroimidazole derivative of 1,2,4‐oxadiazole [24] **2F** demonstrated significant targeted activity against *Clostridioides difficile* while it is inactive against typical gut flora. The potential of oxadiazoles is also emerging as a promising tool for the treatment of neurodegenerative disorders such as Alzheimer's disease (AD), the leading cause of dementia. These compounds can interfere with various aspects of AD pathogenesis, such as the amyloid cascade hypothesis attributing AD to beta‐amyloid (A*β*) plaques and tau protein tangles, the cholinergic hypothesis linking it to a decline in acetylcholine transmission, and the infection hypothesis suggests chronic infections triggering immune activation and inflammation [25, 26, 27]. Both 1,2,4‐ and 1,3,4‐oxadiazole derivatives have demonstrated inhibition of cholinesterases (ChE), as seen in the case of the selective butyrylcholinesterase (BChE) 1,2,4‐oxadiazole‐based inhibitor [28] **2G**, and the pyridine‐thiazol‐1,3,4‐oxadiazole hybrids [29], represented by the most potent derivative **2H** (Figure 2). With growing emphasis on the development of multitarget‐directed ligands (MTDLs) in AD therapy, oxadiazole containing molecules have been developed that combine ChE inhibition with other neuroprotective mechanisms. For example, 5‐(pyridine‐3‐yl)‐1,3,4‐oxadiazoles [30], represented by lead compound **2I**, demonstrate dual acetylcholinesterase (AChE) and BChE inhibition activity, antioxidant properties, and ability to decrease A*β*_1‐42_ levels in experimental models (Figure 2).

Considering the well‐documented relevance of oxadiazole, pyridine, and quinoline scaffolds, as well as the importance of nitro group in medicinal chemistry, we aimed to incorporate them into a single molecule to improve antimicrobial, ChE inhibitory, and antioxidant potency via molecular hybridisation approach. In this study, we present a series of unsymmetrically substituted 1,3,4‐ and 1,2,4‐oxadiazole derivatives containing at least one nitro group, along with their synthetic precursors, and evaluation of their biological activities.

## 2 Results and Discussion

### 2.1 Chemistry

The synthesis of targeted nitro‐substituted 1,3,4‐ and 1,2,4‐oxadiazoles involved a multistep process.

**Scheme 1:**
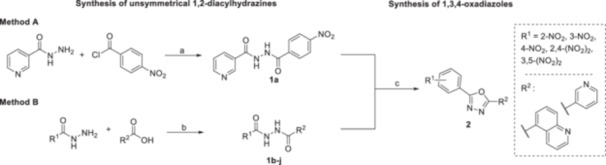
Synthesis of diacylhydrazines **1** and 1,3,4‐oxadiazoles **2**. Reagents and conditions: **a** – K_2_CO_3_, THF, 0°C, 2 h, 76%; **b** – EDC*HCl, HOBt, DMF, 0°C → rt, overnight, 42%–88%; **c** – TsCl, Et_3_N, CH_2_Cl_2_, rt, 3 h, 26%–81%.

For 1,3,4‐oxadiazoles **2**, the procedure began with the synthesis of unsymmetrical 1,2‐diacylhydrazines **1** from hydrazides, followed by cyclization to desired oxadiazoles (Scheme 1). Diacylhydrazines **1** were prepared using two methods. The first method (**Method A**), used only for one compound (**1a**), was the direct acylation of nicotinohydrazide with 4‐nitrobenzoyl chloride in the presence of potassium carbonate as a base, providing 76% yield. The second (**Method B**), more widely used approach in this study involved carbodiimide‐mediated coupling of hydrazides with carboxylic acids in *N*,*N*‐dimethylformamide (DMF) using 1‐hydroxybenzotriazole (HOBt) hydrate as an additive. This method provided yields ranging from 42% to 88%, with no clear relationship between yields and the nature of the nitro acids or hydrazides used. The preparation of cyclic analogues **2** was achieved via dehydrative cyclization of 1,2‐diacylhydrazines **1** mediated by *p*‐toluenesulfonyl chloride (TsCl) in the presence of triethylamine as a base. The yields for these compounds varied from 26% to 81%. Quinoline derivatives provided generally lower isolated yields than pyridyl ones (26%–75% and 31%–81%, respectively). Column chromatography was used for purification and identified as a key factor in the overall yield as it contributed to product losses.

**Scheme 2:**
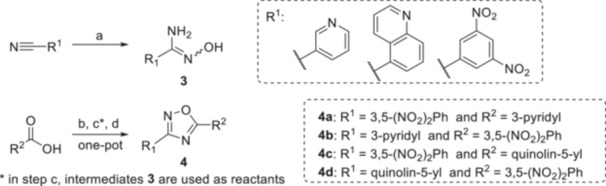
Synthesis of 1,2,4‐oxadiazoles **4** from intermediates **3**. Reagents and conditions: **a** – NH_2_OH*HCl, *N*,*N*‐diisopropylethylamine, THF, 70°C, 3 h; **b** – CDI, 30 min, DMSO, rt; **c** – appropriate intermediate **3**, rt, overnight; **d** – powdered NaOH, rt, 2 h.

In the case of 1,2,4‐oxadiazoles (Scheme 2), nitrile starting materials were converted to unavailable amidoximes. This first step was accomplished by reacting with a mild excess of hydroxylamine hydrochloride and *N*,*N*‐diisopropylethylamine (DIPEA) under heating in tetrahydrofuran (THF). Yields for this step were 56% and 97%, favouring the *N*′‐hydroxy‐3,5‐dinitrobenzimidamide over its quinoline analogue. These amidoxime intermediates were then coupled with carboxylic acids using carbonyldiimidazole (CDI) in dimethyl sulphoxide (DMSO), followed by treatment with sodium hydroxide to form the 1,2,4‐oxadiazoles **4**. The final products were isolated after column chromatography. Here, yields were consistently lower than for 1,3,4‐oxadiazoles, ranging from 40% to 51%.

The electron‐withdrawing nature of the nitro groups and, notably, the steric effect associated with 2,4‐dinitrophenyl moiety likely reduced the nucleophilicity of key intermediates, thus impacting the overall reaction efficiency. Despite these challenges, the methods provided sufficient amounts and purity of the targeted products for subsequent biological evaluations, with extensive use of column chromatography and extractions for purification.

### 2.2 Biological Activity

#### 2.2.1 Inhibition of AChE and BChE

The activity of the compounds against AChE and BChE was evaluated spectrophotometrically using modified Ellman's method [31], which is widely recognised for in vitro screening of potential cholinesterase inhibitors. The method indirectly quantifies enzymatic activity by detecting 5‐mercapto‐2‐nitrobenzoic acid ion, formed through the reaction of thiol reagent 5,5′‐dithiobis(2‐nitrobenzoic acid) (DTNB) with thiocholine, a hydrolytic product generated from the cleavage of acetylthiocholine (ATCh) and butyrylthiocholine (BTCh) catalysed by AChE and BChE, respectively [32]. AChE used in this study was derived from electric eel, while BChE was sourced from equine serum. Inhibitory activities are expressed as concentrations that cause 50% inhibition of enzyme activity (IC_50_). Furthermore, selectivity indexes (SI), which quantify the selectivity of the compounds for AChE over BChE, were calculated as the ratio of IC_50_ for BChE/IC_50_ for AChE. The registered drugs rivastigmine, a dual pseudo‐irreversible acylating inhibitor of AChE and BChE, and a competitive (non‐covalent) inhibitor donepezil, were used as reference molecules for comparison. The results of the evaluation are shown in Table 1 (for Compounds **1** and **2**) and Table 2 (for Compounds **3** and **4**).

**Table 1:** Inhibition of cholinesterases of Compounds **1** and **2** and their selectivity indexes (SI).

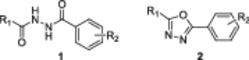					
Compound	R_1_	R_2_	AChE	BChE	SI
			IC_50_ (µM)	IC_50_ (µM)	IC_50_ BChE/IC_50_ AChE
**1a**	3‐Pyridyl	4‐NO_2_	50.16 ± 1.13	170.27 ± 6.82	3.4
**1b**	3‐Pyridyl	3‐NO_2_	41.18 ± 1.56	200.67 ± 3.07	4.9
**1c**	3‐Pyridyl	2‐NO_2_	69.41 ± 3.96	179.45 ± 6.63	2.6
**1d**	3‐Pyridyl	2,4‐diNO_2_	53.40 ± 1.83	380.63 ± 7.08	7.1
**1e**	3‐Pyridyl	3,5‐diNO_2_	51.29 ± 0.45	347.65 ± 35.13	6.8
**1f**	Quinolin‐5‐yl	4‐NO_2_	62.67 ± 2.27	288.91 ± 5.29	4.6
**1g**	Quinolin‐5‐yl	3‐NO_2_	58.88 ± 4.09	230.90 ± 4.40	3.9
**1h**	Quinolin‐5‐yl	2‐NO_2_	50.01 ± 1.02	197.08 ± 7.78	3.9
**1i**	Quinolin‐5‐yl	2,4‐diNO_2_	92.00 ± 5.26	398.44 ± 8.48	4.3
**1j**	Quinolin‐5‐yl	3,5‐diNO_2_	37.67 ± 1.89	232.59 ± 3.65	6.2
**2a**	3‐Pyridyl	4‐NO_2_	12.13 ± 0.23	**85.86** ± **1.83**	7.1
**2b**	3‐Pyridyl	3‐NO_2_	**6.68** ± **0.12**	101.68 ± 2.06	**15.2**
**2c**	3‐Pyridyl	2‐NO_2_	19.46 ± 0.12	137.73 ± 6.47	7.1
**2d**	3‐Pyridyl	2,4‐diNO_2_	**7.15** ± **0.40**	322.32 ± 5.55	**45.1**
**2e**	3‐Pyridyl	3,5‐diNO_2_	**1.47** ± **0.05**	140.82 ± 6.21	**95.8**
**2f**	Quinolin‐5‐yl	4‐NO_2_	59.88 ± 3.93	233.29 ± 4.93	3.9
**2g**	Quinolin‐5‐yl	3‐NO_2_	20.66 ± 0.73	174.65 ± 9.09	8.5
**2h**	Quinolin‐5‐yl	2‐NO_2_	30.36 ± 1.30	187.81 ± 1.37	6.2
**2i**	Quinolin‐5‐yl	2,4‐diNO_2_	29.21 ± 0.04	346.08 ± 6.94	**11.8**
**2j**	Quinolin‐5‐yl	3,5‐diNO_2_	**2.64** ± **0.02**	> 500	**> 189.4**
**Donepezil**	—	—	0.015 ± 0.0001	0.031 ± 0.001	2.1
**Rivastigmine**	—	—	56.10 ± 1.41	38.40 ± 1.97	0.7

*Note:* The best activities are highlighted in bold.

Abbreviations: AChE, acetylcholinesterase; BChE, butyrylcholinesterase.

**Table 2:** Inhibition of cholinesterases of Compounds **3** and **4** and their selectivity indexes (SI).

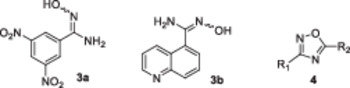					
Compound	R_1_	R_2_	AChE	BChE	SI
			IC_50_ (µM)	IC_50_ (µM)	IC_50_ BChE/IC_50_ AChE
**3a**	—	—	26.37 ± 0.09	**65.05** ± **3.42**	2.5
**3b**	—	—	63.26 ± 2.82	**60.93** ± **3.25**	1.0
**4a**	3,5‐dinitrophenyl	3‐pyridyl	22.07 ± 1.09	150.75 ± 3.91	6.8
**4b**	3‐pyridyl	3,5‐dinitrophenyl	**18.26** ± **0.09**	**45.09** ± **0.44**	2.5
**4c**	3,5‐dinitrophenyl	quinolin‐5‐yl	**15.46** ± **0.24**	223.12 ± 11.75	**14.4**
**4d**	quinolin‐5‐yl	3,5‐dinitrophenyl	33.72 ± 2.00	123.03 ± 9.89	3.6
**Donepezil**	—	—	0.015 ± 0.0001	0.031 ± 0.001	2.1
**Rivastigmine**	—	—	56.10 ± 1.41	38.40 ± 1.97	0.7

*Note:* The best activities are highlighted in bold.

Abbreviations: AChE, acetylcholinesterase; BChE, butyrylcholinesterase.

Diacylhydrazines **1** inhibited AChE with IC_50_ values ranging from 37.67 to 92.0 µM, indicating better potency against AChE than BChE. The most potent compound in this series was the quinoline derivative bearing 3,5‐dinitrobenzoyl group (**1j**). Overall, it is ambiguous whether nicotinohydrazide‐based or quinoline‐carbohydrazide‐based derivatives are better. An analogous conclusion was found for analysing the number of nitro groups on the acyl moieties and their positions. Generally, the inhibitory potency of these compounds appears to depend on the structure of both acyl fragments. Seven compounds exhibited comparable activity to rivastigmine, while two were less active, and two were more potent. Although 1,2‐diacylhydrazines **1** preferentially inhibited AChE, the inhibition was not entirely selective, with SI ranging from 2.6 to 7.1. This indicates that BChE was also inhibited at higher concentrations (IC_50_ values between 170.27 and 398.44 µM). However, none of the compounds reached the potency of rivastigmine for this enzyme. In this case, nicotinohydrazide‐based compounds showed slightly improved efficacy than quinoline‐based derivatives. Regarding nitro substitution, monosubstituted nitro derivatives were generally more effective than disubstituted ones, with the 2‐nitro position being preferred.

1,3,4‐Oxadiazoles **2**, with one exception, were significantly more active than their diacylhydrazine precursors **1**, exhibiting IC_50_ values for AChE of 1.47–59.88 µM, in some cases, such as the most potent 3,5‐dinitrophenyl derivative (**2e**), demonstrating a 35‐fold increase in activity. In this series, the derivatives prepared from nicotinohydrazide were unequivocally more effective than those from quinoline‐5‐carbohydrazide. The influence of nitro groups was also more consistent, favouring 3,5‐dinitrophenyl scaffold, followed by 3‐nitro derivative, and then isomeric 2,4‐dinitro compounds. With one exception, all these oxadiazoles were more potent than rivastigmine, with one derivative being up to 38× more effective, which is remarkable. All 1,3,4‐oxadiazoles **2** demonstrated more potent AChE inhibition, and five of them exhibited a high selectivity (SI ≥ 11.8), with the most selective being the 3,5‐dinitro quinoline derivative **2j** (SI > 189.4), which had IC_50_ value for BChE exceeding 500 µM. The lowest reached IC_50_ value for BChE was 85.86 µM (**2a**). As noted, except for one inactive compound **2j**, cyclization led to at least a modest improvement in *in vitro* inhibitory activity. The nicotinohydrazide‐based derivatives were again more effective, and monosubstitution with a nitro group was generally preferred. However, the activity of all the compounds against BChE remained substantially lower than that of rivastigmine.

Both amidoximes **3** inhibited BChE at relatively low concentrations of 60.93 and 65.05 µM, while their activity against AChE varied significantly. The 3,5‐dinitrophenyl derivative **3a** was notably more potent than the quinoline **3b** (26.37 vs. 63.26 µM) and even more effective than rivastigmine. The latter derivative exhibited the most balanced inhibition of AChE and BChE within this study, with SI of 1.0, indicating no preference between the two ChE.

The cyclization of amidoximes **3** to 1,2,4‐oxadiazoles **4** resulted in compounds with significantly reduced activity against BChE in three cases (IC_50_ ═ 123.03–223.12 µM). However, one molecule (**4b**) substituted with 5‐(3‐pyridyl) and 3‐(3,5‐dinitrophenyl) exhibited the most potent BChE inhibition in the series (IC_50_ ═ 45.09 µM). Regarding AChE inhibition, the synthesis of 1,2,4‐oxadiazoles **4** proved successful, as all compounds demonstrated improved efficacy compared to their amidoxime precursors **3**, with IC_50_ values ranging from 15.46 to 33.72 µM. Pyridyl derivatives exhibited a narrower concentration range; however, a thorough structure‐activity relationship (SAR) analysis considering the substituents, and their positions was limited by the small number of compounds and conflicting conclusions. Compound **4c** emerged as a selective AChE inhibitor (SI ═ 14.4), while other SI values ranged from 2.5 to 6.8. All the compounds shared superior AChE inhibition compared to rivastigmine, but reverse behaviour was observed for BChE inhibition.

In general, 1,3,4‐oxadiazoles **2** generally outperformed their diacylhydrazine precursors **1** regarding cholinesterases inhibition. This can be attributed to the enhanced rigidity and planarity of the oxadiazole ring, which likely promotes better binding within the active AChE site. Among the oxadiazoles, 1,3,4‐regioisomers **2** were more potent than 1,2,4‐oxadiazoles **4**. This difference may arise from the distinct orientation of nitrogen atoms in the ring structure, which influences hydrogen bonding and interactions with residues in the AChE active site. In contrast to rivastigmine, none of the tested compounds exceeded the inhibitory potency of the reference drug donepezil.

Overall, SAR analysis highlights that structural rigidity, electron‐withdrawing substituents, and choice of heterocyclic core (pyridine vs. quinoline) significantly influence the inhibitory activity of these compounds.

#### 2.2.2 Antioxidant Activity

Several studies have established a strong link between oxidative stress and A*β* pathology, as A*β* induces oxidative stress and contributes to its deposition through oxidative mechanisms. A*β* increases the production of free radicals and reactive oxygen species (ROS), leading to oxidative stress [33, 34]. ROS, in turn, cause the oxidation of biomolecules, resulting in cellular damage [35]. The presence of oxidative stress in AD is evidenced by elevated levels of protein oxidation and nitration products, advanced glycosylation, lipid peroxidation, and the formation of toxic species such as peroxides, alcohols, free carbonyl compounds, and oxidative modifications in DNA [36, 37, 38]. Antioxidants have demonstrated significant potential in mitigating the symptoms of AD [39], however, their therapeutic potential is controversial [36]. Given that ROS play an important role in neurotoxicity in AD, developing compounds with dual action, both antioxidants and cholinesterase inhibitors, represents a promising strategy for neuroprotection.

Antioxidants also play a crucial role in infectious diseases by modulating the oxidative stress associated with infection. Oxidative stress arises when ROS and reactive nitrogen species (RNS) are produced in excess, overwhelming the natural antioxidant defences. During infections, immune cells generate ROS and RNS as a defence mechanism to kill invading pathogens. However, this same oxidative stress can lead to tissue damage, then inflammation, and, in severe cases, contribute to the progression of the disease, e.g., in the case of tuberculosis or hepatitis C. Antioxidants, therefore, offer potential therapeutic benefits by reducing this oxidative stress and protecting tissues, though the balance between supporting host defence and preventing tissue damage is delicate [40, 41, 42].

That is why we investigated the antioxidant capacity of four selected promising derivatives (1,3,4‐oxadiazoles **2a**, **2b**, and **2d**, and 1,2,4‐oxadiazole **4b**). Two 3,5‐dinitro compounds were excluded due to solubility issues. Total antioxidant capacity (TAC) was determined using a commercially available Antioxidant Assay Kit [42], which is suitable for determining antioxidant activity in various biological samples (e.g., serum, plasma, urine), as well as in food and beverages. This kit can also be utilised in drug discovery and pharmacology to study the effect of drugs on TAC. The Antioxidant Assay Kit measures TAC by reducing Cu(II) ions by an antioxidant to Cu(I) ion, forming a coloured complex with dye reagent. The colour intensity, measured at 570 nm, is directly proportional to TAC in the sample. The kit has a linear detection range from 1.5 to 1000 µM Trolox equivalents.

As the antioxidant activity was primarily considered an “adjunctive” activity to ChE inhibition, we used the concentrations based on IC_50_ values for AChE. The first concentration used matched IC_50_ values, while the second was ten times higher. Results are summarised in Table 3.

**Table 3:** Antioxidant activity.

Compound	Concentration used (µM)	TAC
**2a**	12.13	22.5
	121.3	**69.0**
**2b**	6.7	21.0
	67	22.5
**2d**	7.15	20.5
	71.5	20.0
**4b**	18.3	**25.0**
	183	64.0

*Note:* The best activities are highlighted in bold.

Abbreviation: TAC, total antioxidant capacity (Trolox equivalents).

All compounds demonstrated a certain capacity to reduce oxidative stress, with TAC values ranging from 20.5 to 25 µM at concentrations corresponding to their IC_50_ values for AChE inhibition. Interestingly, two compounds (**2d** and **2b**) did not exhibit enhanced antioxidant activity at higher concentrations. In contrast, 1,3,4‐oxadiazole **2a** and 1,2,4‐oxadiazole **4b** were more potent at higher concentrations, reaching TAC values of 69.0 and 64.0 µM, respectively.

In conclusion, the compounds displayed moderate but notable antioxidant properties that are concentration‐dependent for some compounds, though not in a strictly linear manner.

#### 2.2.3 Antimycobacterial Activity

The evaluated nitro‐containing oxadiazoles and their precursors demonstrated varying levels of activity against different mycobacterial species, including both drug‐susceptible *Mycobacterium tuberculosis* (*Mtb*) and non‐tuberculous mycobacteria (NTM) such as *Mycobacterium avium* and *Mycobacterium kansasii* (Table 4).

**Table 4:** Antimycobacterial activity.

Compound	MIC (µM)						
	*Mtb* 331/88		*M. avium* 330/88		*M. kansasii* 235/80		
	14 days	21 days	14 days	21 days	7 days	14 days	21 days
**1e**	125	125	> 1000	> 1000	> 1000	> 1000	> 1000
**1i**	1000	> 1000	> 1000	> 1000	500	500	500
**2b**	250	> 500	> 500	> 500	> 500	> 500	> 500
**2c**	1000	1000	> 1000	> 1000	500	1000	1000
**2d**	**2**	**2**	> 1000	> 1000	> 1000	> 1000	> 1000
**2e**	**2**	**2**	**32**	**32**	**4**	**8**	**8**
**2i**	4	**8**	> 250	> 250	> 250	> 250	> 250
**2j**	**4**	**8**	> 250	> 250	> 250	> 250	> 250
**Isoniazid**	0.5	0.5	> 250	> 250	2	2	2

*Note:* The best activities are highlighted in bold.

Abbreviations: *M. avium*, *Mycobacterium avium*; *M. kansasii*, *Mycobacterium kansasii*; *Mtb*, *Mycobacterium tuberculosis*.

Among precursors, only 2,4‐dinitro **1i** and 3,5‐dinitro **1e** derivatives showed notable antimycobacterial activity, while six 1,3,4‐oxadiazoles (**2b**‐**2e**, **2i**, and **2j**) exhibited significant antimycobacterial effects. The most potent compounds were those containing 2,4‐dinitrophenyl (**2d** and **2i**) and 3,5‐dinitrophenyl (**2e** and **2j**) groups, favouring 3‐pyridyl analogues **2d** and **2e** over quinoline‐5‐yl counterparts (**2i**, **2j**). These compounds inhibited selectively *Mtb* with MIC values of 2 µM and 4–8 µM, respectively. Only 1,3,4‐oxadiazole **2e** also inhibited *M. kansasii* at low concentrations of 4–8 µM, and, uniquely, also chemoresistant *M. avium* at 32 µM. Derivative **2b** exhibited a mild and selective bacteriostatic effect against *Mtb* after 14 days of incubation. The precursor **1i** and the oxadiazole **2c** showed dual inhibition of both *Mtb* and *M. kansasii*, but only at comparatively high concentrations (≥ 500 µM).

Overall, 1,3,4‐oxadiazoles **2** were more potent than their diacylhydrazine precursors **1**, which is consistent with the previous study [43] and can be considered valuable pharmacophores for antimycobacterial activity. However, their MIC values were higher than those of the reference drug INH.

#### 2.2.4 Antibacterial and Antifungal Activity

The synthesised derivatives were evaluated for antibacterial and antifungal activity based on the known antimicrobial activity of various oxadiazoles and nitro group‐containing compounds. Initially, the compounds were tested against one representative Gram‐positive strain (methicillin‐susceptible *Staphylococcus aureus*; *SA*) and one Gram‐negative strain (*Escherichia coli;* EC). Further testing was performed against six additional bacterial strains if significant activity was observed. Compounds **2f–2h** could not be evaluated due to their limited solubility in DMSO or precipitation in the test medium. Among the tested compounds, only 2‐(2,4‐dinitrophenyl)‐5‐(pyridin‐3‐yl)‐1,3,4‐oxadiazole (**2d**) and its 3,5‐dinitro isomer **2e** demonstrated notable antibacterial activity and was further evaluated against a broader panel comprising four Gram‐positive cocci and four Gram‐negative bacteria. MIC values of the most active compound **2d** were particularly low against methicillin‐resistant *S. aureus* (MRSA) and *Staphylococcus epidermidis* (7.81–31.25 µM), while for SA and *Enterococcus*, significantly higher values (125–250 µM) were recorded (Table 5). Gram‐negative pathogens showed complete resistance. Interestingly, MRSA was more susceptible to **2d** compared to SA. However, overall, MIC values of **2d** were not superior to those of the reference antibiotic piperacillin. 3,5‐Dinitro analogue **2e** exhibited similar activity against *S. aureus* and *Enterococcus*, although it was slightly less active against MRSA and *S. epidermidis*. On the other hand, it inhibited all Gram‐negative pathogens at a uniform concentration of 250 µM.

**Table 5:** Antibacterial activity.

	MIC (µM)							
Strain	SA		MRSA		SE		EF	
incubation	24 h	48 h	24 h	48 h	24 h	48 h	24 h	48 h
**2d**	125	250	**15.62**	**31.25**	**7.81**	**15.62**	250	250
**2e**	250	250	250	250	31.25	62.5	250	250
**PIP**	3.7		29.6		0.23		3.7	

*Note:* The best activities are highlighted in bold.

Abbreviations: EF, *Enterococcus faecalis*; MRSA, methicillin‐resistant *S. aureus*; PIP, piperacillin; SA, methicillin‐susceptible *Staphylococcus aureus*; SE, *Staphylococcus epidermidis*.

The compounds were initially tested against a representative yeast strain (*Candida albicans*) and one filamentous fungus strain (*Trichophyton interdigitale*; TI) for antifungal activity screening. Compounds **2f–2h** could not be assessed again due to solubility limitations. Consistently with the antibacterial testing, only compound **2d** demonstrated antifungal activity, but it was limited to TI with MIC of 125 µM. However, this activity level was deemed insufficient for further evaluation against the remaining planned six fungal strains. None of the other compounds demonstrated antifungal efficacy.

#### 2.2.5 Cytotoxicity Studies

To complete the biological activity profile, we also evaluated the cytotoxicity of the selected derivatives against a panel of mammalian cell lines, including both tumorous and non‐tumorous cells. The panel comprised A2058 (human melanoma), Calu‐1 (human lung carcinoma), HepG2 (human hepatocellular carcinoma), MonoMac‐6 (human monocytic leukaemia), HT‐29 (human colorectal adenocarcinoma), Caco‐2 (human colorectal adenocarcinoma), and Vero E6 (African green monkey kidney epithelial) cell lines.

Three highly active oxadiazoles (and their corresponding acyclic precursors) were selected for this evaluation: compound **2d**, exhibiting broad‐spectrum antimicrobial activity (bacteria, mycobacteria, and fungi), and compounds **2e** and **2j**, which share potent antimycobacterial activity combined with significant enzyme inhibition. Among 1,2,4‐oxadiazoles, the inhibitor **4b** was selected.

The results (Table 6) indicate low cytotoxicity of the investigated derivatives toward both normal and cancer cell lines, supporting a favourable safety profile and suggesting that their antimicrobial activity is selective rather than a consequence of general non‐specific toxicity.

**Table 6:** Cytotoxicity of the selected nitro derivatives **1**, **2**, and **4**.

Compound	A2058	Calu‐1	HepG2	MonoMac‐6	HT‐29	Caco‐2	Vero
	IC_50_ [µM]	IC_50_ [µM]	IC_50_ [µM]	IC_50_ [µM]	IC_50_ [µM]	IC_50_ [µM]	IC_50_ [µM]
**1d**	> 100	> 100	> 100	> 100	> 100	> 100	> 100
**1e**	> 100	> 100	> 100	> 100	> 100	> 100	> 100
**1j**	> 100	> 100	> 100	> 100	> 100	> 100	> 100
**2d**	> 100	> 100	> 100	> 100	> 100	> 100	> 100
**2e**	89.2	> 100	76.2	78.2	100	> 100	> 100
**2j**	> 100	> 100	> 100	> 100	> 100	> 100	> 100
**4b**	> 100	> 100	> 100	> 100	> 100	> 100	> 100

*Note:* The best activities are highlighted in bold.

### 2.3 Molecular Docking

A molecular modelling study was performed to elucidate the possible orientation of the ligands in the active site of AChE. For this purpose, the most potent inhibitors in the 1,3,4‐oxadiazole series **2e** and **2j** were selected as representatives, and their resulting conformation in the active site gorge was investigated.

**Figure 3:**
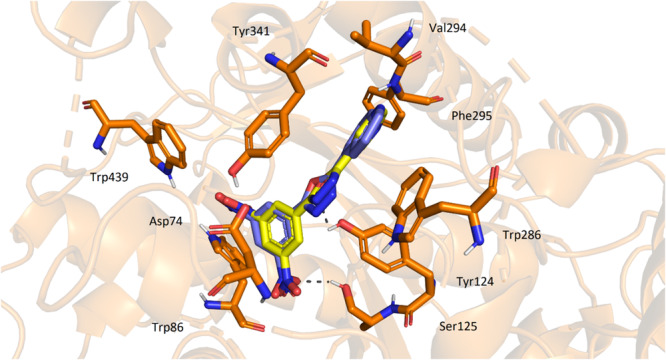
Position of ligands **2e** (yellow) and **2j** (blue) in the active site of acetylcholinesterase (AChE) with interacting amino acid residues (orange).

Both ligands' top‐scored docking poses showed similar orientations and interactions with amino acid residues. Both molecules were located near the entrance to the active site gorge with their 3‐pyridyl and quinoline‐5‐yl moieties directed out of the enzyme, showing π‐π interactions with Trp286 and Tyr341. These amino acid residues form the so‐called peripheral anionic site (PAS), and at least part of the inhibitors' effect can therefore be attributed to their interactions with this region. 3‐Pyridyl moiety of **2e** displayed additional H‐bond interaction with Phe295. 3,5‐Dinitrophenyl moieties were stored deeper within the cavity and were further stabilised by interactions with Trp86, Ser125, and Trp439. Central 1,3,4‐oxadiazole part of both ligands indicated H‐bond with Tyr124 (Figure 3).

**Figure 4:**
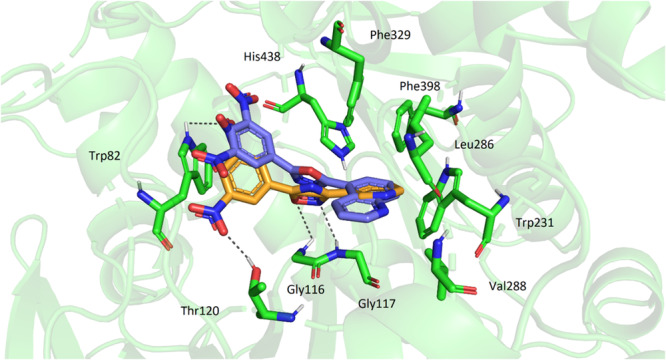
A comparison of **4b** (orange) and **2j** (blue) conformations in the active site of butyrylcholinesterase (BChE; green).

The compound **2j**, one of the most effective AChE inhibitors, showed very low activity against BChE. Its resulting conformation in BChE was compared to that of ligand **4b**, for which the highest inhibitory activity against BChE was reported. Both molecules were positioned relatively deep within the active site gorge with their quinoline‐5‐yl and 3‐pyridyl moieties occupying similar space, surrounded by aromatic and hydrophobic residues, namely Trp231, Leu286, Val288, Phe329, and Phe398, forming π–π or hydrophobic interactions. The rest of the **4b** molecule was slightly shifted compared to **2j**. This resulted in additional H‐bond formation between **4b** and residues Thr120, Gly116, and Gly117. Other amino acid residues that indicated interactions with both molecules were Trp82 and His438 (Figure 4).

The docking results indicate that the described binding mode is unique and clearly distinct from that of clinically used drugs, as the presented oxadiazoles simultaneously interact with PAS and bind to amino acid residues located inside the enzyme cavity, yet without engaging all key amino acid residues of either the catalytic triad or the catalytic anionic site (CAS). Consequently, their mechanism differs from that of rivastigmine, a covalent inhibitor that acylates the active‐site serine, as well as from donepezil, which dually interacts with both PAS and CAS, and from galantamine, which primarily targets the catalytic triad.

## 3 Conclusion

The study highlights the promising potential of nitrophenyl group‐containing 1,3,4‐ and 1,2,4‐oxadiazole derivatives and their synthetic precursors substituted additionally with heteroaryl (3‐pyridyl, quinoline‐5‐yl) in medicinal chemistry, particularly as enzyme inhibitors, antimicrobials, and antioxidants.

The synthesised compounds exhibited dual inhibitory activity against both AChE and BChE with low micromolar IC_50_ values and competitive inhibition based on molecular docking. Adjunct antioxidant activities underline their potential suitability for the development of compounds targeting neurodegenerative diseases. Some compounds also exhibited potent antimicrobial activity, mainly against *M. tuberculosis* and staphylococci. The bioactivity can be modified based on a specific substitution pattern. In general, 1,3,4‐oxadiazoles **3** were superior to their 1,2,4‐analogues **4** and acyclic 1,2‐diacylhydrazine precursors **1**. The oxadiazoles can interfere with AD pathogenesis by multiple actions: inhibition of ChE, antioxidant (excessive production of ROS), and antimicrobial properties (infectious hypothesis of AD).

The integration of nitro groups into these oxadiazole scaffolds not only enhanced bioactivity but also opened avenues for further molecular hybridisation strategies. The promising results suggest that future research should expand the structural diversity of 2,5‐disubstituted 1,3,4‐oxadiazoles, exploring their potential as multitarget‐directed ligands.

## 4 Experimental

### 4.1 Chemistry

#### 4.1.1 General

All chemicals used were purchased from Merck (Darmstadt, Germany), VWR/Avantor (Stříbrná Skalice, Czech Republic), Apollo Scientific Ltd. (Stockport, United Kingdom), and Lach‐Ner (Neratovice, Czech Republic), and were used without further purification. Reaction progress was monitored, and the prepared compounds' retention factors (R_f_) were determined using thin‐layer chromatography (TLC). As the stationary phase, TLC plates coated with a 0.2 mm layer of silica gel Merck 60 F254, purchased from Merck Millipore (Darmstadt, Germany), were used. The mobile phase (MP) for both TLC and column chromatography consisted of a mixture of dichloromethane (DCM) and methanol (MeOH) in volumetric ratios of 93:7 for *N*′‐acyl nicotinic hydrazides (**1a–e**), *N*′‐acyl quinoline‐5‐carbohydrazides (**1f–j**), and amidoximes **3**, and 97:3 for 1,3,4‐ and 1,2,4‐oxadiazoles (**2** and **4**). The stationary phase for column chromatography was Merck Kieselgel 60 Å silica gel (0.040–0.063 mm), also purchased from Merck (Darmstadt, Germany).

**Figure 5:**
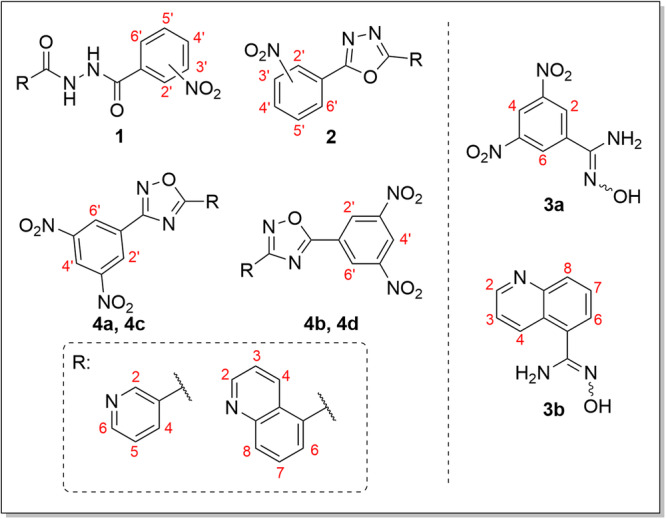
The general numbering scheme used for the assignment of the ^1^H NMR signals of compounds **1**–**4**.

The structures of the synthesised compounds were confirmed using nuclear magnetic resonance (NMR) spectroscopy. Both proton and carbon spectra were recorded at ambient temperature or 60°C in deuterated dimethyl sulphoxide (DMSO‐*d_6_*) on Varian VNMR S500 instrument (500 MHz for ^1^H and 126 MHz for ^13^C; Varian Comp., Palo Alto, USA) and JEOL JNM‐ECZ 600 R (600 MHz for ^1^H and 151 MHz for ^13^C spectra; JEOL, Tokyo, Japan). Chemical shift values (*δ*) are expressed in parts per million (ppm). They are indirectly referenced to tetramethylsilane in proton spectra, while in carbon spectra, they were determined based on the central line of the solvent (DMSO‐*d*_*6*_ at *δ* ═ 39.5 ppm). Coupling constants (*J*) are given in Hz. NMR spectra were analysed using MestReNova software (Mestrelab Research, Santiago de Compostela, Spain). The general numbering scheme used for the assignment of the ^1^H NMR signals of compounds **1**–**4** is shown in Figure 5. Copies of ^1^H and ^13^C NMR spectra of the prepared compounds are provided in the Supporting Information (Figures S1–S52).

Melting points were determined using open glass capillaries in Melting Point Machine B‐540 (Büchi, Flawil, Switzerland). The resulting values are reported as a range and are uncorrected. Infrared (IR) spectra were obtained using a Nicolet 6700 FT‐IR spectrometer (Thermo Fisher Scientific, Waltham, MA, USA) over the range of 600–4000 cm⁻¹, utilising the attenuated total reflectance (ATR) technique with a germanium crystal. The intensities of the IR bands are denoted as follows: very strong (vs), strong (s), medium (m), weak (w), and broad (br). Elemental composition was analysed using a Vario MICRO Cube Elemental Analyzer (Elementar Analysensysteme, Hanau, Germany), with calculated and experimental values reported as percentages.

#### 4.1.2 General Procedure for the Synthesis of 1,2‐Diacylhydrazines (**1a**–**j**)

**Method A** (used for the synthesis of compound **1a**): 1 mmol of nicotinohydrazide (137 mg) was suspended with two equivalents of potassium carbonate (2 mmol, 276 mg) in 15 mL of anhydrous THF. The mixture was stirred while cooling to 0°C in an ice bath, and then 1.1 equivalents of 4‐nitrobenzoyl chloride (1.1 mmol, 204 mg) were gradually added. The reaction mixture was allowed to react until an acceptable conversion rate was achieved (monitored by TLC). After 2 h, THF was evaporated, the resulting product was dissolved in demineralised water, and the pH was adjusted to approximately 6 by adding 0.5 M hydrochloric acid. Extraction was performed using ethyl acetate. After the first extraction, the product precipitated in the organic layer. Subsequently, the aqueous phase was separated, and ethyl acetate fraction was filtered off, and the final product was dried.

**Method B** (used for the synthesis of all compounds **1** except **1a**): 2 mmol of corresponding carboxylic acid, 1 equivalent of corresponding hydrazide (2 mmol), and 1.1 equivalents of HOBt (2.2 mmol, 297 mg) were suspended together in 20 mL of DMF. The reaction mixture was stirred and cooled to 0°C in an ice bath. To the cooled mixture, 1.3 equivalents of *N*‐(3‐dimethylaminopropyl)‐*N*′‐ethylcarbodiimide hydrochloride (EDC*HCl; 2.6 mmol, 498 mg) were added. The progress of the reaction was monitored by TLC, and after achieving an acceptable conversion rate (in most cases, ca. 2 h), the mixture was evaporated. The crude product was dissolved in ethyl acetate and extracted. For **1c**, **1e**, and **1j**, after extraction with water and brine, the organic layer was dried over sodium sulphate and concentrated to initiate crystallisation. The suspension was filtered, and the resulting product was dried. For **1b**, **1d**, and **1f–1i**, the products precipitated in the organic phase after the first extraction, so the aqueous phase was separated, and the organic phase was filtered, and products were dried. If necessary, the next step was the purification of these compounds: **1d** was recrystallised using a hexane/ethyl acetate mixture; **1g** was washed with acetone, in which the product was insoluble, and then dried; compound **1f** was purified by column chromatography (MP: DCM/MeOH 93:7), the combined fractions were evaporated, and the product was suspended in hexane, filtered, and dried.

##### 4.1.2.1 *N*′‐(4‐Nitrobenzoyl)nicotinoylhydrazide (**1a**)

White crystalline solid; yield 76% (method A); mp: 275.5°C–277.0°C (254°C−255°C [44]). **IR (ATR):** ṽ ═ 3203 (br; NH), 1610 (s; C═O), 1588 (vs; v_as_ NO_2_), 1350 (vs; ν_s_ NO_2_) cm^−1^. ^**1**^ **H NMR** (600 MHz, DMSO‐*d*_*6*_): *δ* 10.96 (d, *J* ═ 1.3 Hz, 1H, NH), 10.87 (d, *J* ═ 1.3 Hz, 1H, NH), 9.10–9.08 (m, 1H, H2), 8.79 (dd, *J* ═ 4.8, 1.7 Hz, 1H, H6), 8.41–8.37 (m, 2H, H3′, H5′), 8.27 (ddd, *J* ═ 7.9, 2.3, 1.7 Hz, 1H, H4), 8.18–8.14 (m, 2H, H2′, H6′), 7.59 (dd, *J* ═ 7.9, 4.8 Hz, 1H, H5). ^**13**^ **C NMR** (151 MHz, DMSO‐*d*_*6*_): *δ* 164.4 (C═O), 164.3 (C═O), 152.6 (Ar‐*C*), 149.5 (Ar‐*C*), 148.4 (Ar‐*C*), 138.0 (Ar‐*C*), 135.3 (Ar‐*C*), 129.0 (Ar‐*C*), 128.0 (Ar‐*C*), 123.8 (Ar‐*C*), 123.7 ppm (Ar‐*C*). **Anal. calcd.** for C_13_H_10_N_4_O_4_ (286.25): C 54.55, H 3.52, N 19.57. Found: C 54.63, H 3.41, N 19.52. **R**_**f**_ (DCM/MeOH 93:7): 0.37.

##### 4.1.2.2 *N*′‐(3‐Nitrobenzoyl)nicotinoylhydrazide (**1b**)

White crystalline solid; yield 86% (method B); mp: 216.1°C–218.7°C. **IR (ATR)**: ṽ ═ 3207 (br; NH), 1620 (m; C═O), 1598 (vs; v_as_ NO_2_), 1348 (vs; ν_s_ NO_2_) cm^−1^. **¹H NMR** (600 MHz, DMSO‐*d*_*6*_): *δ* 11.03 (s, 1H, NH), 10.89 (s, 1H, NH), 9.09 (d, *J* ═ 2.1 Hz, 1H, H2), 8.79 (dd, *J* ═ 4.8, 1.9 Hz, 1H, H6), 8.76 (t, *J* ═ 2.2 Hz, 1H, H2′), 8.47 (dd, *J* ═ 8.1, 2.3 Hz, 1H, H4′), 8.37 (d, *J* ═ 8.0 Hz, 1H, H6′), 8.28 (dt, *J* ═ 7.9, 2.1 Hz, 1H, H4), 7.86 (t, *J* ═ 8.0 Hz, 1H, H5′), 7.59 (dd, *J* ═ 7.9, 4.8 Hz, 1H, H5). **¹³C NMR** (151 MHz, DMSO‐*d*_*6*_): *δ* 164.4 (C═O), 163.9 (C═O), 152.6 (Ar‐*C*), 148.5 (Ar‐*C*), 147.9 (Ar‐*C*), 135.3 (Ar‐*C*), 133.8 (Ar‐*C*), 133.7 (Ar‐*C*), 130.5 (Ar‐*C*), 128.0 (Ar‐*C*), 126.6 (Ar‐*C*), 123.7 (Ar‐*C*), 122.2 ppm (Ar‐*C*). **Anal. calcd.** for C_13_H_10_N_4_O_4_ (286.25): C 54.55, H 3.52, N 19.57. Found: C 54.62, H 3.47, N 19.66. **R**_**f**_ (DCM/MeOH 93:7): 0.35.

##### 4.1.2.3 *N*′‐(2‐Nitrobenzoyl)nicotinoylhydrazide (**1c**)

White crystalline solid; yield 51% (method B); mp: 193.1°C–196.5°C. **IR (ATR)**: ṽ ═ 3239 (br; NH), 1650 (vs; C═O), 1524 (vs; v_as_ NO_2_), 1349 (vs; ν_s_ NO_2_) cm^−1^. **¹H NMR** (600 MHz, DMSO‐*d*_*6*_): *δ* 10.96 (s, 1H, NH), 10.79 (s, 1H, NH), 9.09 (d, *J* ═ 2.1 Hz, 1H, H2), 8.78 (dd, *J* ═ 4.8, 1.7 Hz, 1H, H6), 8.29 (dt, *J* ═ 7.9, 2.1 Hz, 1H, H4), 8.13–8.09 (m, 1H, H3′), 7.89 (t, *J* ═ 7.4 Hz, 1H, H6′), 7.81–7.76 (m, 2H, H4′, H5′), 7.57 (dd, *J* ═ 7.9, 4.8 Hz, 1H, H5). **¹³C NMR** (151 MHz, DMSO‐*d*_*6*_): *δ* 164.8 (C═O), 164.2 (C═O), 152.6 (Ar‐*C*), 148.6 (Ar‐*C*), 147.3 (Ar‐*C*), 135.3 (Ar‐*C*), 133.8 (Ar‐*C*), 131.6 (Ar‐*C*), 130.2 (Ar‐*C*), 129.6 (Ar‐*C*), 127.9 (Ar‐*C*), 124.4 (Ar‐*C*), 123.7 ppm (Ar‐*C*). **Anal. calcd.** for C_13_H_10_N_4_O_4_ (286.25): C 54.55, H 3.52, N 19.57. Found: C 54.64, H 3.47, N 19.51. **R**_**f**_ (DCM/MeOH 93:7): 0.33.

##### 4.1.2.4 *N*′‐(2,4‐Dinitrobenzoyl)nicotinoylhydrazide (**1d**)

Pinkish crystalline solid; yield 42% (method B); mp: 231.4°C–233.1°C. **IR (ATR)**: ṽ ═ 3234 (br; NH), 1654 (s; C═O), 1532 (vs; v_as_ NO_2_), 1353 (s; ν_s_ NO_2_) cm⁻¹. **¹H NMR** (600 MHz, DMSO‐*d*_*6*_): *δ* 11.07 (s, 2H, CO–NH–NH–CO), 9.12–9.06 (m, 1H, H2), 8.83 (d, *J* ═ 2.3 Hz, 1H, H3′), 8.79 (d, *J* ═ 4.8 Hz, 1H, H6), 8.71 (dd, *J* ═ 8.3, 2.3 Hz, 1H, H5′), 8.29 (d, *J* ═ 7.9 Hz, 1H, H4), 8.03 (d, *J* ═ 8.3 Hz, 1H, H6′), 7.58 (dd, *J* ═ 7.9, 4.8 Hz, 1H, H5). **¹³C NMR** (151 MHz, DMSO‐*d*_*6*_): *δ* 164.2 (C═O), 163.4 (C═O), 152.7 (Ar‐*C*), 148.6 (Ar‐*C*), 148.4 (Ar‐*C*), 147.2 (Ar‐*C*), 135.4 (Ar‐*C*), 135.2 (Ar‐*C*), 131.3 (Ar‐*C*), 128.4 (Ar‐*C*), 127.8 (Ar‐*C*), 123.7 (Ar‐*C*), 120.0 ppm (Ar‐*C*). **Anal. calcd.** for C_13_H_9_N_5_O_6_ (331.24): C 47.14, H 2.74, N 21.14. Found: C 47.22, H 2.85, N 21.27. **R**_**f**_ (DCM/MeOH 93:7): 0.33.

##### 4.1.2.5 *N*′‐(3,5‐Dinitrobenzoyl)nicotinohydrazide (**1e**)

Beige crystalline solid; yield 85% (method B); mp: 262°C–264°C. **IR (ATR)**: ṽ ═ 3175 (br; NH), 1660 (vs; C═O), 1537 (vs; v_as_ NO_2_), 1349 (s; v_s_ NO_2_) cm^−1^. ^**1**^ **H NMR** (500 MHz, DMSO‐*d*_*6*_): *δ* 11.55 (s, 1H, NH), 11.34 (s, 1H, NH), 9.26 (dd, *J* ═ 2.1, 0.8 Hz, 1H, H2), 9.14 (d, *J* ═ 2.1 Hz, 2H, H2′, H6′), 9.03 (t, *J* ═ 2.1 Hz, 1H, H4′), 8.96 (dd, *J* ═ 5.2, 1.7 Hz, 1H, H6), 8.65 (dt, *J* ═ 8.1, 1.7 Hz, 1H, H4), 7.89 (ddd, *J* ═ 8.1, 5.2, 0.8 Hz, 1H, H5). ^**13**^ **C NMR** (126 MHz, DMSO‐*d*_*6*_): *δ* 163.0 (C═O), 161.9 (C═O), 149.2 (Ar‐*C*), 148.4 (Ar‐*C*), 145.5 (Ar‐*C*), 139.4 (Ar‐*C*), 134.6 (Ar‐*C*), 129.2 (Ar‐*C*), 127.8 (Ar‐*C*), 125.4 (Ar‐*C*), 121.7 ppm (Ar‐*C*). **Anal. calcd.** for C_13_H_9_N_5_O_6_ (331.24): C 47.14, H 2.74, N 21.14. Found: C 47.50, H 2.91, N 21.01. **R**_**f**_ (DCM/MeOH 93:7): 0.2.

##### 4.1.2.6 *N*′‐(4‐Nitrobenzoyl)quinoline‐5‐carbohydrazide (**1f**)

White crystalline solid; yield 46% (method B); mp: 258.9°C–260.7°C. **IR (ATR)**: ṽ ═ 3193 (br; NH), 1616 (s; C═O), 1574 (vs; v_as_ NO_2_), 1351 (s; v_s_ NO_2_) cm^−1^. **¹H NMR** (600 MHz, DMSO‐*d*_*6*_): *δ* 11.05 (s, 1H, NH), 10.74 (s, 1H, NH), 9.00 (dd, *J* ═ 4.2, 1.7 Hz, 1H, H2), 8.84 (dd, *J* ═ 8.7, 1.7 Hz, 1H, H4), 8.44–8.35 (m, 2H, H3′, H5′), 8.25–8.16 (m, 3H, H6, H7, H8), 7.92–7.82 (m, 2H, H2′, H6′), 7.67 (dd, *J* ═ 8.7, 4.2 Hz, 1H, H3). **¹³C NMR** (151 MHz, DMSO‐*d*_*6*_): *δ* 166.9 (C═O), 164.3 (C═O), 151.1 (Ar‐*C*), 149.5 (Ar‐*C*), 147.5 (Ar‐*C*), 137.9 (Ar‐*C*), 133.6 (Ar‐*C*), 132.6 (Ar‐*C*), 131.7 (Ar‐*C*), 129.1 (Ar‐*C*), 128.6 (Ar‐*C*), 126.2 (Ar‐*C*), 125.3 (Ar‐*C*), 123.8 (Ar‐*C*), 122.2 ppm (Ar‐*C*). **Anal. calcd.** for C_17_H_12_N_4_O_4_ (336.31): C 60.71, H 3.60, N 16.66. Found: C 60.85, H 3.68, N 16.51. **R**_**f**_ (DCM/MeOH 93:7): 0.55.

##### 4.1.2.7 *N*′‐(3‐Nitrobenzoyl)quinoline‐5‐carbohydrazide (**1g**)

White crystalline solid; yield 49% (method B); mp: 245.8°C–247.9°C. **IR (ATR)**: ṽ ═ 3188 (br; NH), 1612 and 1600 (s; C═O), 1568 (vs; v_as_ NO_2_), 1348 (vs; v_s_ NO_2_) cm^−1^. **¹H NMR** (600 MHz, DMSO‐*d*_*6*_): *δ* 11.12 (s, 1H, NH), 10.75 (s, 1H, NH), 9.02–8.98 (m, 1H, H2), 8.87–8.78 (m, 2H, H4, H2′), 8.48 (d, *J* ═ 8.3 Hz, 1H, H4′), 8.42 (d, *J* ═ 7.8 Hz, 1H, H6′), 8.20 (d, *J* ═ 7.6 Hz, 1H, H5′), 7.91–7.85 (m, 3H, H6, H7, H8), 7.68 (dd, *J* ═ 8.7, 4.1 Hz, 1H, H3). **¹³C NMR** (151 MHz, DMSO‐*d*_*6*_): *δ* 167.0 (C═O), 163.9 (C═O), 151.1 (Ar‐*C*), 147.9 (Ar‐*C*), 147.5 (Ar‐*C*), 133.9 (Ar‐*C*), 133.7 (Ar‐*C*), 133.6 (Ar‐*C*), 132.6 (Ar‐*C*), 131.7 (Ar‐*C*), 130.5 (Ar‐*C*), 128.7 (Ar‐*C*), 126.7 (Ar‐*C*), 126.2 (Ar‐*C*), 125.3 (Ar‐*C*), 122.3 (Ar‐*C*), 122.3 ppm (Ar‐*C*). **Anal. calcd.** for C_17_H_12_N_4_O_4_ (336.31): C 60.71, H 3.60, N 16.66. Found: C 60.88, H 3.72, N 16.53. **R**_**f**_ (DCM/MeOH 93:7): 0.40.

##### 4.1.2.8 *N*′‐(2‐Nitrobenzoyl)quinoline‐5‐carbohydrazide (**1h**)

White crystalline solid; yield 88% (method B); mp: 268.6°C–269.5°C. **IR (ATR)**: ṽ ═ 3173 (br; NH), 1612 and 1602 (s; C═O), 1573 (vs; v_as_ NO_2_), 1355 (s; v_s_ NO_2_) cm^−1^. **¹H NMR** (600 MHz, DMSO‐*d*_*6*_): *δ* 10.87 (s, 1H, NH), 10.83 (s, 1H, NH), 8.99 (dd, *J* ═ 4.1, 1.8 Hz, 1H, H2), 8.78 (dd, *J* ═ 8.7, 1.8 Hz, 1H, H4), 8.19 (dd, *J* ═ 7.5, 2.3 Hz, 1H, H3′), 8.13 (d, *J* ═ 8.3 Hz, 1H, H6′), 7.93–7.89 (m, 1H, H5′), 7.87–7.81 (m, 3H, H6, H7, H8), 7.84–7.77 (m, 1H, H4′), 7.66 (dd, *J* ═ 8.7, 4.1 Hz, 1H, H3). **¹³C NMR** (151 MHz, DMSO‐*d*_*6*_): *δ* 166.8 (C═O), 164.9 (C═O), 151.0 (Ar‐*C*), 147.5 (Ar‐*C*), 147.3 (Ar‐*C*), 133.8 (Ar‐*C*), 133.6 (Ar‐*C*), 132.4 (Ar‐*C*), 131.6 (Ar‐*C*), 130.2 (Ar‐*C*), 129.6 (Ar‐*C*), 128.6 (Ar‐*C*), 128.3 (Ar‐*C*), 126.4 (Ar‐*C*), 125.3 (Ar‐*C*), 124.4 (Ar‐*C*), 122.2 ppm (Ar‐*C*). **Anal. calcd.** for C_17_H_12_N_4_O_4_ (336.31): C 60.71, H 3.60, N 16.66. Found: C 60.82, H 3.73, N 16.56. **R**_**f**_ (DCM/MeOH 93:7): 0.43.

##### 4.1.2.9 *N*′‐(2,4‐Dinitrobenzoyl)quinoline‐5‐carbohydrazide (**1i**)

Red crystalline solid; yield 78% (method B); mp: 271.8°C–273.5°C. **IR (ATR)**: ṽ ═ 3176 (br; NH), 1655 (s; C═O), 1532 (vs; v_as_ NO_2_), 1350 (vs; v_s_ NO_2_) cm^−1^. **¹H NMR** (600 MHz, DMSO‐*d*_*6*_): *δ* 11.15 (s, 1H, NH), 10.95 (s, 1H, NH), 8.99 (dd, *J* ═ 4.1, 1.7 Hz, 1H, H2), 8.85 (d, *J* ═ 2.4 Hz, 1H, H3′), 8.76 (dd, *J* ═ 8.8, 1.7 Hz, 1H, H4), 8.72 (dd, *J* ═ 8.3, 2.4 Hz, 1H, H5′), 8.22–8.17 (m, 1H, H8), 8.07 (d, *J* ═ 8.3 Hz, 1H, H6′), 7.88–7.84 (m, 2H, H6, H7), 7.66 (dd, *J* ═ 8.8, 4.1 Hz, 1H, H3). **¹³C NMR** (151 MHz, DMSO‐*d*_*6*_): *δ* 166.7 (C═O), 163.5 (C═O), 151.1 (Ar‐*C*), 148.5 (Ar‐*C*), 147.5 (Ar‐*C*), 147.3 (Ar‐*C*), 135.2 (Ar‐*C*), 133.5 (Ar‐*C*), 132.1 (Ar‐*C*), 131.8 (Ar‐*C*), 131.3 (Ar‐*C*), 128.7 (Ar‐*C*), 128.5 (Ar‐*C*), 126.5 (Ar‐*C*), 125.3 (Ar‐*C*), 122.3 (Ar‐*C*), 120.0 ppm (Ar‐*C*). **Anal. calcd.** for C_17_H_11_N_5_O_6_ (381.30): C 53.55, H 2.91, N 18.37. Found: C 53.63, H 2.85, N 18.45. **R**_**f**_ (DCM/MeOH 93:7): 0.39.

##### 4.1.2.10 *N*′‐(3,5‐Dinitrobenzoyl)quinoline‐5‐carbohydrazide (**1j**)

Beige crystalline solid; yield 76% (method B); mp: 275°C–276°C. **IR (ATR)**: ṽ ═ 3215 (br; NH), 1675 (s; C═O), 1537 (vs; v_as_ NO_2_), 1350 (vs; v_s_ NO_2_) cm^−1^. ^**1**^ **H NMR** (600 MHz, DMSO‐*d*_*6*_): *δ* 11.62 (s, 1H, NH), 11.11 (s, 1H, NH), 9.25 (dd, *J* ═ 4.7, 1.6 Hz, 1H, H2), 9.23–9.20 (m, 1H, H4), 9.18 (d, *J* ═ 2.1 Hz, 2H, H2′, H6′), 9.05 (t, *J* ═ 2.1 Hz, 1H, H4′), 8.49–8.45 (m, 1H, H8), 8.14–8.07 (m, 2H, H6, H7), 8.02 (dd, *J* ═ 8.6, 4.7 Hz, 1H, H3). ^**13**^ **C NMR** (151 MHz, DMSO‐*d*_*6*_): *δ* 166.0 (C═O), 162.0 (C═O), 148.4 (Ar‐*C*), 148.1 (Ar‐*C*), 142.1 (Ar‐*C*), 139.2 (Ar‐*C*), 134.6 (Ar‐*C*), 132.5 (Ar‐*C*), 131.5 (Ar‐*C*), 128.0 (Ar‐*C*), 127.8 (Ar‐*C*), 127.2 (Ar‐*C*), 125.7 (Ar‐*C*), 122.9 (Ar‐*C*), 121.7 ppm (Ar‐*C*). **Anal. calcd.** for C_17_H_11_N_5_O_6_ (381.30): C 53.55, H 2.91, N 18.37. Found: C 53.80, H 3.17, N 18.00. **R**_**f**_ (DCM/MeOH 93:7): 0.2.

#### 4.1.3 General Procedure for the Synthesis of 1,3,4‐oxadiazoles (**2a**–**j**)

The synthesis of 1,3,4‐oxadiazoles **3** was carried out according to a uniform procedure [45]: 1 equivalent of 1,2‐diacylhydrazine **1** (1 mmol) was suspended in 50 mL of DCM. While stirring, 3 equivalents of TsCl (3 mmol, 572 mg) were added to the mixture, followed by the dropwise addition of 5 equivalents of triethylamine (5 mmol, 697 µL). The reaction mixture was allowed to proceed until an acceptable conversion was achieved, as indicated by TLC. Further processing varied between nicotinic and quinoline derivatives.

For **2a–2e**, volatiles were evaporated. The crude products were suspended in ethyl acetate, and the precipitate of triethylammonium chloride was filtered off. The filtrate was evaporated to dryness. These crude products were then purified using column chromatography (gradient elution, MP: DCM to DCM/MeOH 97:3). The combined fractions were evaporated, and the products were suspended in hexane. The resulting products were filtered and dried.

For **2f–2j**, the reaction mixture was extracted with demineralised water, a saturated solution of ammonium chloride, and saturated brine. The organic phase was dried over sodium sulphate, filtered, and evaporated to dryness. The crude products were purified using column chromatography (MP: DCM/MeOH 97:3). The combined fractions were evaporated, and the crude products were suspended in hexane. The resulting products were filtered and dried.

##### 4.1.3.1 2‐(4‐Nitrophenyl)‐5‐(pyridin‐3‐yl)‐1,3,4‐oxadiazole (**2a**)

White crystalline solid; yield 46%; mp: 212.8°C–215.3°C (222°C–224°C [46]). **IR (ATR)**: ṽ ═ 1515 (s; v_as_ NO_2_), 1340 (vs; v_s_ NO_2_) cm^−1^. **¹H NMR** (600 MHz, DMSO‐*d₆*): *δ* 9.34 (dd, *J* ═ 2.2, 0.9 Hz, 1H, H2), 8.85 (dd, *J* ═ 4.8, 1.7 Hz, 1H, H6), 8.54 (ddd, *J* ═ 7.9, 2.2, 1.7 Hz, 1H, H4), 8.47–8.45 (m, 2H, H3′, H5′), 8.44–8.41 (m, 2H, H2′, H6′), 7.69 (ddd, *J* ═ 7.9, 4.8, 0.9 Hz, 1H, H5). **¹³C NMR** (151 MHz, DMSO‐*d₆*): *δ* 163.1 (Ar‐*C*), 163.1 (Ar‐*C*), 152.8 (Ar‐*C*), 149.3 (Ar‐*C*), 147.6 (Ar‐*C*), 134.5 (Ar‐*C*), 128.7 (Ar‐*C*), 128.2 (Ar‐*C*), 124.6 (Ar‐*C*), 124.4 (Ar‐*C*), 119.7 ppm (Ar‐*C*). **Anal. calcd.** for C_13_H_8_N_4_O_3_ (268.23): C 58.21, H 3.01, N 20.89. Found: C 58.33, H 3.14, N 20.94. **R**_**f**_ (DCM/MeOH 97:3): 0.6.

##### 4.1.3.2 2‐(3‐Nitrophenyl)‐5‐(pyridin‐3‐yl)‐1,3,4‐oxadiazole (**2b**)

White crystalline solid; yield 31%; mp: 185.7°C–187.4°C (192°C [47]). **IR (ATR)**: ṽ ═ 1523 (vs; v_as_ NO_2_), 1354 (vs; v_s_ NO_2_) cm^−1^. **¹H NMR** (600 MHz, DMSO‐*d₆*): *δ* 9.36 (d, *J* ═ 2.3 Hz, 1H, H2), 8.86 (t, *J* ═ 2.0 Hz, 1H, H2′), 8.84 (dd, *J* ═ 4.9, 1.7 Hz, 1H, H6), 8.59 (dt, *J* ═ 8.0, 1.9 Hz, 1H, H4′), 8.56 (dt, *J* ═ 7.9, 2.0 Hz, 1H, H6′), 8.49 (ddd, *J* ═ 8.1, 2.3, 1.7 Hz, 1H, H4), 7.95 (t, *J* ═ 8.0 Hz, 1H, H5′), 7.69 (dd, *J* ═ 8.1, 4.8 Hz, 1H, H5). **¹³C NMR** (151 MHz, DMSO‐*d₆*): *δ* 162.9 (Ar‐*C*), 162.9 (Ar‐*C*), 152.7 (Ar‐*C*), 148.3 (Ar‐*C*), 147.6 (Ar‐*C*), 134.5 (Ar‐*C*), 132.9 (Ar‐*C*), 131.3 (Ar‐*C*), 126.6 (Ar‐*C*), 124.7 (Ar‐*C*), 124.4 (Ar‐*C*), 121.3 (Ar‐*C*), 119.7 ppm (Ar‐*C*). **Anal. calcd.** for C_13_H_8_N_4_O_3_ (268.23): C 58.21, H 3.01, N 20.89. Found: C 58.35, H 3.12, N 20.97. **R**_**f**_ (DCM/MeOH 97:3): 0.52.

##### 4.1.3.3 2‐(2‐Nitrophenyl)‐5‐(pyridin‐3‐yl)‐1,3,4‐oxadiazole (**2c**)

White‐yellow crystalline solid; yield 41%; mp: 116.4°C–118.1°C (89°C [48]). **IR (ATR)**: ṽ ═ 1524 (vs; v_as_ NO_2_), 1351 (vs; v_s_ NO_2_) cm^−1^. **¹H NMR** (600 MHz, DMSO‐*d₆*): *δ* 9.24 (d, *J* ═ 2.3 Hz, 1H, H2), 8.85 (dd, *J* ═ 4.8, 1.6 Hz, 1H, H6), 8.43 (dt, *J* ═ 8.0, 2.0 Hz, 1H, H3′), 8.23 (dd, *J* ═ 7.9, 1.6 Hz, 1H, H4), 8.20 (dd, *J* ═ 7.6, 1.7 Hz, 1H, H6′), 7.99 (td, *J* ═ 7.7, 1.7 Hz, 1H, H5′), 7.96 (td, *J* ═ 7.7, 1.7 Hz, 1H, H4′), 7.69 (dd, *J* ═ 7.9, 4.8 Hz, 1H, H5). **¹³C NMR** (151 MHz, DMSO‐*d₆*): *δ* 163.1 (Ar‐*C*), 161.0 (Ar‐*C*), 152.9 (Ar‐*C*), 147.9 (Ar‐*C*), 147.4 (Ar‐*C*), 134.4 (Ar‐*C*), 133.7 (Ar‐*C*), 133.6 (Ar‐*C*), 131.4 (Ar‐*C*), 124.8 (Ar‐*C*), 124.5 (Ar‐*C*), 119.5 (Ar‐*C*), 116.7 ppm (Ar‐*C*). **Anal. calcd.** for C_13_H_8_N_4_O_3_ (268.23): C 58.21, H 3.01, N 20.89. Found: C 58.35, H 3.16, N 20.95. **R**_**f**_ (DCM/MeOH 97:3): 0.43.

##### 4.1.3.4 2‐(2,4‐Dinitrophenyl)‐5‐(pyridin‐3‐yl)‐1,3,4‐oxadiazole (**2d**)

Yellow crystalline solid; yield 42%; mp: 178.5°C–181.1°C. **IR (ATR)**: ṽ ═ 1543 and 1524 (vs; v_as_ NO_2_), 1352 and 1341 (s, v_s_ NO_2_) cm^−1^. **¹H NMR** (500 MHz, DMSO‐*d₆*): *δ* 9.27 (dd, *J* ═ 2.2, 0.9 Hz, 1H, H2), 9.02 (d, *J* ═ 2.3 Hz, 1H, H3′), 8.87 (dd, *J* ═ 4.8, 1.7 Hz, 1H, H6), 8.74 (ddd, *J* ═ 8.6, 2.3, 0.9 Hz, 1H, H5′), 8.53 (d, *J* ═ 8.6 Hz, 1H, H6′), 8.47 (ddd, *J* ═ 8.0, 2.3, 1.7 Hz, 1H, H4), 7.70 (ddt, *J* ═ 8.0, 4.8, 0.9 Hz, 1H, H5). **¹³C NMR** (126 MHz, DMSO‐*d₆*): *δ* 163.6 (Ar‐*C*), 159.6 (Ar‐*C*), 153.2 (Ar‐*C*), 149.5 (Ar‐*C*), 147.9 (Ar‐*C*), 147.6 (Ar‐*C*), 134.6 (Ar‐*C*), 132.6 (Ar‐*C*), 127.7 (Ar‐*C*), 124.5 (Ar‐*C*), 121.1 (Ar‐*C*), 120.1 (Ar‐*C*), 119.3 ppm (Ar‐*C*). **Anal. calcd.** for C_13_H_7_N_5_O_5_ (313.23): C 49.85, H 2.25, N 22.36. Found: C 49.92, H 2.11, N 22.47. **R**_**f**_ (DCM/MeOH 97:3): 0.53.

##### 4.1.3.5 2‐(3,5‐Dinitrophenyl)‐5‐(pyridin‐3‐yl)‐1,3,4‐oxadiazole (**2e**)

Beige crystalline solid; yield 81%; mp: 163°C–165°C. **IR (ATR)**: ṽ ═ 1546 and 1535 (s; v_as_ NO_2_), 1347 (vs; v_s_ NO_2_) cm^−1^. ^**1**^ **H NMR** (600 MHz, DMSO‐*d₆*): *δ* 9.44 (dd, *J* ═ 2.3, 0.9 Hz, 1H, H2), 9.21 (d, *J* ═ 2.1 Hz, 2H, H2′, H6′), 9.03 (t, *J* ═ 2.1 Hz, 1H, H4′), 8.86 (dd, *J* ═ 4.8, 1.7 Hz, 1H, H6), 8.64 (ddd, *J* ═ 8.0, 2.3, 1.7 Hz, 1H, H4), 7.70 (ddd, *J* ═ 8.0, 4.8, 0.9 Hz, 1H, H5). ^**13**^ **C NMR** (151 MHz, DMSO‐*d₆*): *δ* 163.5 (Ar‐*C*), 161.8 (Ar‐*C*), 153.0 (Ar‐*C*), 148.8 (Ar‐*C*), 147.8 (Ar‐*C*), 134.7 (Ar‐*C*), 126.6 (Ar‐*C*), 126.0 (Ar‐*C*), 124.4 (Ar‐*C*), 121.2 (Ar‐*C*), 119.5 ppm (Ar‐*C*). **Anal. calcd.** for C_13_H_7_N_5_O_5_ (313.23): C 49.85, H 2.25, N 22.36. Found: C 50.01, H 2.46, N 22.21. **R**_**f**_ (DCM:MeOH 97:3): 0.3.

##### 4.1.3.6 2‐(4‐Nitrophenyl)‐5‐(quinolin‐5‐yl)‐1,3,4‐oxadiazole (**2f**)

Yellowish crystalline solid; yield 26%; mp: 222.2°C–224.4°C. **IR (ATR)**: ṽ ═ 1531 (vs; v_as_ NO_2_), 1339 (vs; v_s_ NO_2_) cm^−1^. **¹H NMR** (600 MHz, DMSO‐*d₆*): *δ* 9.54 (dd, *J* ═ 8.7, 1.6 Hz, 1H, H2), 9.05 (dd, *J* ═ 4.1, 1.6 Hz, 1H, H4), 8.50 (dd, *J* ═ 7.5, 1.0 Hz, 1H, H8), 8.47–8.44 (m, 2H, H3′, H5′), 8.44–8.41 (m, 2H, H2′, H6′), 8.31 (dd, *J* ═ 7.4, 1.0 Hz, 1H, H6), 7.99 (t, *J* ═ 7.5 Hz, 1H, H7), 7.77 (dd, *J* ═ 8.7, 4.1 Hz, 1H, H3). **¹³C NMR** (151 MHz, DMSO‐*d₆*): *δ* 164.0 (Ar‐*C*), 162.4 (Ar‐*C*), 151.4 (Ar‐*C*), 149.3 (Ar‐*C*), 147.7 (Ar‐*C*), 133.9 (Ar‐*C*), 133.8 (Ar‐*C*), 129.3 (Ar‐*C*), 129.0 (Ar‐*C*), 128.8 (Ar‐*C*), 128.2 (Ar‐*C*), 124.8 (Ar‐*C*), 124.6 (Ar‐*C*), 123.2 (Ar‐*C*), 119.8 ppm (Ar‐*C*). **Anal. calcd.** for C_17_H_10_N_4_O_3_ (318.29): C 64.15, H 3.17, N 17.60. Found: C 64.29, H 3.07, N 17.73. **R**_**f**_ (DCM/MeOH 97:3): 0.52.

##### 4.1.3.7 2‐(3‐Nitrophenyl)‐5‐(quinolin‐5‐yl)‐1,3,4‐oxadiazole (**2g**)

White crystalline solid; yield 62%; mp: 230.9°C–232.6°C. **IR (ATR)**: ṽ ═ 1524 (vs; v_as_ NO_2_), 1351 (vs; v_s_ NO_2_) cm^−1^. **¹H NMR** (600 MHz, DMSO‐*d₆*): *δ* 9.59 (dd, *J* ═ 8.7, 1.5 Hz, 1H, H2), 9.08 (dd, *J* ═ 4.1, 1.6 Hz, 1H, H4), 8.90 (t, *J* ═ 1.6 Hz, 1H, H2′), 8.64 (dt, *J* ═ 7.9, 1.0 Hz, 1H, H6′), 8.59 (dd, *J* ═ 7.4, 1.2 Hz, 1H, H6), 8.52 (dd, *J* ═ 8.2, 2.2 Hz, 1H, H4′), 8.34 (d, *J* ═ 8.2 Hz, 1H, H8), 8.02 (dd, *J* ═ 8.5, 7.3 Hz, 1H, H7), 7.98 (t, *J* ═ 8.0 Hz, 1H, H5′), 7.81 (dd, *J* ═ 8.7, 4.1 Hz, 1H, H3). **¹³C NMR** (151 MHz, DMSO‐*d₆*): *δ* 163.5 (Ar‐*C*), 162.1 (Ar‐*C*), 151.0 (Ar‐*C*), 147.5 (Ar‐*C*), 133.4 (Ar‐*C*), 133.4 (Ar‐*C*), 132.6 (Ar‐*C*), 131.0 (Ar‐*C*), 128.9 (Ar‐*C*), 128.6 (Ar‐*C*), 126.0 (Ar‐*C*), 124.7 (Ar‐*C*), 124.6 (Ar‐*C*), 122.7 (Ar‐*C*), 121.0 (Ar‐*C*), 119.7 (Ar‐*C*), 117.0 ppm (Ar‐*C*). **Anal. calcd.** for C_17_H_10_N_4_O_3_ (318.29): C 64.15, H 3.17, N 17.60. Found: C 64.26, H 3.04, N 17.68. **R**_**f**_ (DCM/MeOH 97:3): 0.52.

##### 4.1.3.8 2‐(2‐Nitrophenyl)‐5‐(quinolin‐5‐yl)‐1,3,4‐oxadiazole (**2h**)

Yellowish crystalline solid; yield 75%; mp: 174.8°C–176.0°C. **IR (ATR)**: ṽ ═ 1522 (vs; v_as_ NO_2_), 1349 (s; v_s_ NO_2_) cm^−1^. **¹H NMR** (500 MHz, DMSO‐*d₆*): *δ* 9.47 (dd, *J* ═ 8.7, 1.7 Hz, 1H, H2), 9.07 (dd, *J* ═ 4.2, 1.7 Hz, 1H, H4), 8.34–8.31 (m, 2H, H8, H6′), 8.27–8.23 (m, 2H, H6, H3′), 8.04–7.94 (m, 3H, H7, H4′, H5′), 7.79 (dd, *J* ═ 8.7, 4.2 Hz, 1H, H3). **¹³C NMR** (126 MHz, DMSO‐*d₆*): *δ* 164.0 (Ar‐*C*), 160.5 (Ar‐*C*), 151.5 (Ar‐*C*), 148.0 (Ar‐*C*), 147.7 (Ar‐*C*), 134.0 (Ar‐*C*), 133.7 (Ar‐*C*), 133.7 (Ar‐*C*), 133.6 (Ar‐*C*), 131.5 (Ar‐*C*), 129.2 (Ar‐*C*), 129.1 (Ar‐*C*), 124.8 (Ar‐*C*), 124.8 (Ar‐*C*), 123.3 (Ar‐*C*), 119.6 (Ar‐*C*), 116.8 ppm (Ar‐*C*). **Anal. calcd.** for C_17_H_10_N_4_O_3_ (318.29): C 64.15, H 3.17, N 17.60. Found: C 64.23, H 3.08, N 17.71. **R**_**f**_ (DCM/MeOH 97:3): 0.52.

##### 4.1.3.9 2‐(2,4‐Dinitrophenyl)‐5‐(quinolin‐5‐yl)‐1,3,4‐oxadiazole (**2i**)

Yellowish crystalline solid; yield 36%; mp: 206.4°C–207.9°C. **IR (ATR)**: ṽ ═ 1545 and 1532 (vs; v_as_ NO_2_), 1353 and 1345 (s; v_s_ NO_2_) cm^−1^. **¹H NMR** (600 MHz, DMSO‐*d₆*): *δ* 9.43 (dd, *J* ═ 8.7, 1.7 Hz, 1H, H2), 9.07 (dd, *J* ═ 4.1, 1.6 Hz, 1H, H4), 8.99 (d, *J* ═ 2.3 Hz, 1H, H3’), 8.74 (dd, *J* ═ 8.5, 2.3 Hz, 1H, H5′), 8.56 (d, *J* ═ 8.5 Hz, 1H, H6′), 8.37–8.33 (m, 2H, H6, H8), 8.03–7.97 (m, 1H, H7), 7.77 (dd, *J* ═ 8.7, 4.1 Hz, 1H, H3). **¹³C NMR** (151 MHz, DMSO‐*d₆*): *δ* 164.3 (Ar‐*C*), 158.9 (Ar‐*C*), 151.2 (Ar‐*C*), 149.3 (Ar‐*C*), 147.8 (Ar‐*C*), 147.5 (Ar‐*C*), 133.9 (Ar‐*C*), 133.2 (Ar‐*C*), 132.5 (Ar‐*C*), 129.0 (Ar‐*C*), 128.7 (Ar‐*C*), 127.4 (Ar‐*C*), 124.7 (Ar‐*C*), 122.9 (Ar‐*C*), 121.2 (Ar‐*C*), 119.8 (Ar‐*C*), 119.2 ppm (Ar‐*C*). **Anal. calcd.** for C_17_H_9_N_5_O_5_ (363.29): C 56.21, H 2.50, N 19.28. Found: C 56.29, H 2.58, N 19.35. **R**_**f**_ (DCM/MeOH 97:3): 0.35.

##### 4.1.3.10 2‐(3,5‐Dinitrophenyl)‐5‐(quinolin‐5‐yl)‐1,3,4‐oxadiazole (**2j**)

Yellow‐brown crystalline solid; yield 56%; mp: 254°C–256°C. **IR (ATR)**: ṽ ═ 1530 (vs; v_as_ NO_2_), 1346 (vs; v_s_ NO_2_) cm^−1^. ^**1**^ **H NMR** (600 MHz, DMSO‐*d₆*): *δ* 9.57 (dd, *J* ═ 8.8, 1.6 Hz, 1H, H2), 9.22 (d, *J* ═ 2.1 Hz, 2H, H2′, H6′), 9.08 (dd, *J* ═ 4.0, 1.6 Hz, 1H, H4), 9.05 (t, *J* ═ 2.1 Hz, 1H, H4′), 8.64 (d, *J* ═ 7.7 Hz, 1H, H8), 8.36 (d, *J* ═ 7.8 Hz, 1H, H6), 8.03 (t, *J* ═ 7.8 Hz, 1H, H7), 7.80 (dd, *J* ═ 8.7, 4.0 Hz, 1H, H3). ^**13**^ **C NMR** (151 MHz, DMSO‐*d₆*): *δ* 164.1 (Ar‐*C*), 161.0 (Ar‐*C*), 151.1 (Ar‐*C*), 148.7 (Ar‐*C*), 147.5 (Ar‐*C*), 133.7 (Ar‐*C*), 133.5 (Ar‐*C*), 129.4 (Ar‐*C*), 128.7 (Ar‐*C*), 126.4 (Ar‐*C*), 126.0 (Ar‐*C*), 124.7 (Ar‐*C*), 122.9 (Ar‐*C*), 120.8 (Ar‐*C*), 119.4 ppm (Ar‐*C*). **Anal. calcd.** for C_17_H_9_N_5_O_5_ (363.29): C 56.21, H 2.50, N 19.28. Found: C 56.48, H 2.71, N 19.04. **R**_**f**_ (DCM/MeOH 97:3): 0.4.

#### 4.1.4 General Procedure for the Synthesis of Amidoximes (**3a**–**b**)

This method was used for the synthesis of commercially unavailable amidoximes: 1 mmol of the corresponding nitrile was dissolved in 10 mL of THF. To this solution, 1.7 equivalents of DIPEA (296 µL), and 1.5 hydroxylamine hydrochloride equivalents (104 mg) were added. The reaction mixture was heated under reflux at 70°C for 3 h. The solvent was then evaporated. The crude product was dissolved in ethyl acetate and extracted with a saturated solution of ammonium chloride and then with brine. The organic phase was dried over sodium sulphate and evaporated to dryness to obtain amidoxime intermediates **3a** and **3b**. If necessary, the product was further purified by column chromatography (gradient elution, MP: DCM → DCM/MeOH 97:3 → pure MeOH).

##### 4.1.4.1 *N*′‐Hydroxy‐3,5‐dinitrobenzimidamide (**3a**)

Yellow crystalline solid; yield 97%; mp: 181.8°C–183.7°C (199°C–201°C [49]). **IR (ATR)**: ṽ ═ 3473 (br; OH), 3379 (br; NH_2_), 1660 (s; C═N), 1536 (vs; v_as_ NO_2_), 1344 (vs; v_s_ NO_2_) cm^−1^. **¹H NMR** (500 MHz, DMSO‐*d₆*): *δ* 10.31 (s, 1H, OH), 8.88 (d, *J* ═ 2.1 Hz, 2H, H2, H6), 8.80 (t, *J* ═ 2.1 Hz, 1H, H4), 6.38 (s, 2H, NH_2_). **¹³C NMR** (126 MHz, DMSO‐*d₆*): *δ* 148.1 (C═N), 147.7 (Ar‐*C*), 136.3 (Ar‐*C*), 125.1 (Ar‐*C*), 118.4 ppm (Ar‐*C*). **Anal. calcd.** for C_7_H_6_N_4_O_5_ (226.15): C 37.18, H 2.67, N 24.77. Found: C 37.25, H 2.73, N 24.65. **R**_**f**_ (DCM/MeOH 93:7): 0.63.

##### 4.1.4.2 *N*’‐Hydroxyquinoline‐5‐carboximidamide (**3b**) [50]

Grey crystalline solid; yield 56%; mp: 184.1°C–185.6°C. **IR (ATR)**: ṽ ═ 3452 (br; OH), 3346 (br; NH_2_), 1656 (vs; C═N) cm^−1^. **¹H NMR** (600 MHz, DMSO‐*d₆*): *δ* 9.78 (s, 1H, OH), 8.91 (dd, *J* ═ 4.3, 1.9 Hz, 1H, H2), 8.73 (dd, *J* ═ 8.7, 2.0 Hz, 1H, H6), 8.05 (dd, *J* ═ 8.4, 1.1 Hz, 1H, H4), 7.79–7.77 (m, 1H, H8), 7.69 (dd, *J* ═ 6.9, 1.1 Hz, 1H, H7), 7.56 (dd, *J* ═ 8.7, 4.2 Hz, 1H, H3), 6.14 (s, 2H, NH_2_). **¹³C NMR** (151 MHz, DMSO‐*d₆*): *δ* 150.5 (C═N), 147.7 (Ar‐*C*), 134.3 (Ar‐*C*), 132.2 (Ar‐*C*), 131.1 (Ar‐*C*), 129.9 (Ar‐*C*), 128.8 (Ar‐*C*), 126.8 (Ar‐*C*), 126.1 (Ar‐*C*), 121.5 ppm (Ar‐*C*). **Anal. calcd.** for C_10_H_9_N_3_O (187.2): C 64.16, H 4.85, N 22.45. Found: C 64.29, H 4.70, N 22.61. **R**_**f**_ (DCM/MeOH 93:7): 0.35.

#### 4.1.5 General Procedure for the Synthesis of 1,2,4‐Oxadiazoles [51] (**4a**–**d**)

To a solution of the corresponding carboxylic acid (1.4 mmol; 1 equivalent) in 5 mL of DMSO, 1.2 equivalents of CDI (1.68 mmol, 272 mg) were added. After 30 min, 1 equivalent of the corresponding commercially available or previously prepared amidoxime **3** was added to the reaction mixture, and the mixture was left stirring overnight at room temperature. Subsequently, 1.2 equivalents of powdered NaOH were quickly added. After 2 h, 30 mL of cold demineralised water was added. The resulting suspension was filtered, and the solid portion was dried. The crude product was dissolved in MeOH, evaporated with the addition of silica gel, and purified by column chromatography (MP: DCM/MeOH 97:3). In the case of compound **4d**, purification was carried out using column chromatography with gradient elution (MP: hexane → hexane/ethyl acetate 7:3).

##### 4.1.5.1 3‐(3,5‐Dinitrophenyl)‐5‐(pyridin‐3‐yl)‐1,2,4‐oxadiazole (**4a**)

Yellow crystalline solid; yield 51%; mp: 163.9°C–165.4°C. **IR (ATR)**: ṽ ═ 1554 (s; v_as_ NO_2_), 1346 (vs; v_s_ NO_2_) cm^−1^. **¹H NMR** (600 MHz, DMSO‐*d₆*): *δ* 9.41 (t, *J* ═ 1.2, 1H, H2), 9.09 (d, *J* ═ 2.1 Hz, 2H, H2′, H6′), 9.03 (t, *J* ═ 2.1 Hz, 1H, H4′), 8.93 (dt, *J* ═ 4.9, 1.6 Hz, 1H, H6), 8.66–8.62 (m, 1H, H4), 7.73 (ddq, *J* ═ 7.9, 4.9, 0.9 Hz, 1H, H5). **¹³C NMR** (151 MHz, DMSO‐*d_6_*): *δ* 175.0 (Ar‐*C*), 165.9 (Ar‐*C*), 154.1 (Ar‐*C*), 148.8 (Ar‐*C*), 148.7 (Ar‐*C*), 135.9 (Ar‐*C*), 128.7 (Ar‐*C*), 126.9 (Ar‐*C*), 124.6 (Ar‐*C*), 121.2 (Ar‐*C*), 119.6 ppm (Ar‐*C*). **Anal. calcd.** for C_13_H_7_N_5_O_5_ (313.23): C 49.85, H 2.25, N 22.36. Found: C 49.92, H 2.38, N 22.49. **R**_**f**_ (DCM/MeOH 97:3): 0.46.

##### 4.1.5.2 5‐(3,5‐Dinitrophenyl)‐3‐(pyridin‐3‐yl)‐1,2,4‐oxadiazole (**4b**)

Beige crystalline solid; yield 45%; mp: 158.1°C–160.9°C (137°C–139°C [52]). **IR (ATR)**: ṽ ═ 1537 (vs; v_as_ NO_2_), 1347 (vs; v_s_ NO_2_) cm^−1^. **¹H NMR** (600 MHz, DMSO‐*d₆*): *δ* 9.31–9.29 (m, 1H, H2), 9.18–9.16 (m, 2H, H2′, H6′), 9.14–9.07 (m, 1H, H4′), 8.83 (dd, *J* ═ 4.6, 1.6 Hz, 1H, H6), 8.50 (dt, *J* ═ 7.9, 2.0 Hz, 1H, H4), 7.66 (dd, *J* ═ 8.0, 4.6 Hz, 1H, H5). **¹³C NMR** (151 MHz, DMSO‐*d₆*): *δ* 172.5 (Ar‐*C*), 166.8 (Ar‐*C*), 152.5 (Ar‐*C*), 148.6 (Ar‐*C*), 147.7 (Ar‐*C*), 134.6 (Ar‐*C*), 127.6 (Ar‐*C*), 125.7 (Ar‐*C*), 124.1 (Ar‐*C*), 122.1 (Ar‐*C*), 121.7 ppm (Ar‐*C*). **Anal. calcd.** for C_13_H_7_N_5_O_5_ (313.23): C 49.85, H 2.25, N 22.36. Found: C 49.98, H 2.37, N 22.46. **R**_**f**_ (DCM/MeOH 97:3): 0.35.

##### 4.1.5.3 3‐(3,5‐Dinitrophenyl)‐5‐(quinolin‐5‐yl)‐1,2,4‐oxadiazole (**4c**)

Yellow crystalline solid; yield 41%; mp: 204.4°C–206.4°C. **IR (ATR)**: ṽ ═ 1550 (s; v_as_ NO_2_), 1343 (vs; v_s_ NO_2_) cm^−1^. **¹H NMR** (600 MHz, DMSO‐*d₆*): *δ* 9.57–9.53 (m, 1H, H2), 9.16 (d, *J* ═ 2.1 Hz, 2H, H2′, H6′), 9.10 (dd, *J* ═ 4.1, 1.6 Hz, 1H, H4), 9.04 (t, *J* ═ 2.1 Hz, 1H, H4’), 8.60 (dd, *J* ═ 7.3, 1.3 Hz, 1H, H8), 8.44–8.40 (m, 1H, H6), 8.03 (dd, *J* ═ 8.4, 7.3 Hz, 1H, H7), 7.86 (dd, *J* ═ 8.7, 4.0 Hz, 1H, H3). **¹³C NMR** (151 MHz, DMSO‐*d₆*): *δ* 175.5 (Ar‐*C*), 165.8 (Ar‐*C*), 151.6 (Ar‐*C*), 148.8 (Ar‐*C*), 147.7 (Ar‐*C*), 135.3 (Ar‐*C*), 133.5 (Ar‐*C*), 130.8 (Ar‐*C*), 129.1 (Ar‐*C*), 128.9 (Ar‐*C*), 127.0 (Ar‐*C*), 125.1 (Ar‐*C*), 123.6 (Ar‐*C*), 121.1 (Ar‐*C*), 119.7 ppm (Ar‐*C*). **Anal. calcd.** for C_17_H_9_N_5_O_5_ (363.29): C 56.21, H 2.50, N 19.28. Found: C 56.29, H 2.62, N 19.20. **R**_**f**_ (DCM/MeOH 97:3): 0.38.

##### 4.1.5.4 5‐(3,5‐Dinitrophenyl)‐3‐(quinolin‐5‐yl)‐1,2,4‐oxadiazole (**4d**)

Light yellow crystalline solid; yield 40%; mp: 246.8°C–248.8°C. **IR (ATR)**: ṽ ═ 1541 (vs; v_as_ NO_2_), 1342 (vs; v_s_ NO_2_) cm^−1^. **¹H NMR** (600 MHz, DMSO‐*d₆*): *δ* 9.24 (dd, *J* ═ 8.7, 1.7 Hz, 1H, H2), 9.20 (d, *J* ═ 2.1 Hz, 2H, H2′, H6′), 9.11 (t, *J* ═ 2.1 Hz, 1H, H4′), 9.04 (dd, *J* ═ 4.1, 1.7 Hz, 1H, H4), 8.45 (dd, *J* ═ 7.3, 1.2 Hz, 1H, H8), 8.30 (dt, *J* ═ 8.4, 1.1 Hz, 1H, H6), 7.98 (dd, *J* ═ 8.4, 7.3 Hz, 1H, H7), 7.73 (dd, *J* ═ 8.7, 4.1 Hz, 1H, H3). **¹³C NMR** (151 MHz, DMSO‐*d₆*): *δ* 171.5 (Ar‐*C*), 168.0 (Ar‐*C*), 150.7 (Ar‐*C*), 148.5 (Ar‐*C*), 147.6 (Ar‐*C*), 133.3 (Ar‐*C*), 132.9 (Ar‐*C*), 129.3 (Ar‐*C*), 128.5 (Ar‐*C*), 127.4 (Ar‐*C*), 125.8 (Ar‐*C*), 125.0 (Ar‐*C*), 122.5 (Ar‐*C*), 122.4 (Ar‐*C*), 121.7 ppm (Ar‐*C*). **Anal. calcd.** for C_17_H_9_N_5_O_5_ (363.29): C 56.21, H 2.50, N 19.28. Found: C 56.27, H 2.65, N 19.19. **R**_**f**_ (DCM/MeOH 97:3): 0.36.

### 4.2 Biological Assays

#### 4.2.1 Inhibition of AChE and BChE

The inhibitory activities for AChE and BChE were quantified by determining IC_50_ values using a spectrophotometric assay based on a modified Ellman's method [31]. The final reaction volume was 2000 µL, with enzyme activity of 0.2 U/mL, substrate concentration of 40 µM for either ATCh or BTCh, and 100 µM of DTNB for all reactions. The tested compounds were dissolved in DMSO and subsequently diluted with demineralised water (conductivity 3 µS, BKG Water Treatment, Hradec Králové, Czech Republic) to a concentration of 1000 µM. Each compound and the reference inhibitors rivastigmine and donepezil were tested at five different concentrations in the final reaction mixture. The final concentration of DMSO in all assays was maintained at 0.2%. All experiments were performed in triplicate. The average values of reaction rates (v₀ for the uninhibited reaction and vᵢ for the inhibited reaction) were used to plot the ratio v₀/vᵢ as a function of inhibitor concentration. IC_50_ values were calculated from corresponding regression equations, where y ═ 2 (based on the IC_50_ definition). All graphical representations of the v₀/vᵢ dependence on inhibitor concentration are provided in Supporting Information (Figures S53–S105). Acetylcholinesterase from electric eel (*Electrophorus electricus*; EeAChE) and butyrylcholinesterase from equine serum (EqBChE) were utilised in the study, with rivastigmine serving as a reference dual enzyme inhibitor and donepezil as a reference AChE selective competitive inhibitor. All enzymes, substrates, donepezil, and rivastigmine were obtained from Merck (Prague, Czech Republic).

#### 4.2.2 Antioxidant Activity Determination

Total antioxidant capacity (TAC) was determined using a commercially available kit, the Antioxidant Assay Kit [42] (Catalogue Number MAK334; Merck, Prague, Czech Republic). This kit measures TAC in which Cu(II) ion is reduced by an antioxidant to Cu(I) ion, which then forms a coloured complex with dye reagent. The colour intensity measured at 570 nm is proportional to the TAC in the sample. The kit has a linear detection range from 1.5 to 1000 µM Trolox equivalents. Trolox standards and reaction mixtures were prepared according to the instructions for the Antioxidant Assay Kit. Trolox (6‐hydroxy‐2,5,7,8‐tetramethylchromane‐2‐carboxylic acid) standards were used in 0, 300, 600, and 1000 µM concentrations. The concentrations of investigated derivatives were chosen based on the determined IC_50_ values for AChE and BChE inhibition. A concentration equal to the IC_50_ value and 10 times higher for a specific derivative was always used. Investigated derivatives and Trolox were diluted with ultrapure water (conductivity 0.057 µS; Barnstead MicroPure Water Purification System, Thermo Scientific). Trolox standards, studied derivatives, and blanks were always assayed in duplicate in separate wells of a transparent, flat bottom 96‐well plate. Each well contained 20 µL of suitably diluted Trolox or tested derivative or ultrapure water in 100 µL of the reaction mixture. After 10 min incubation at room temperature, the absorbance was measured at 570 nm on BioTek Synergy H1 Multimode Reader (Agilent). The dependence of absorbance A570 on the concentration of Trolox standard was plotted, and the slope of the standard curve was determined (Supporting Information: Figure S106). The Total Antioxidant Capacity of investigated derivatives was calculated as follows:

$$\text{TAC}\;\left(\mathrm{µM}\right)═\frac{{\left(A_{570}\right)}_{\text{sample}}-{\left(A_{570}\right)}_{\text{blank}}}{\text{slope}\;({\mathrm{µM}}^{-1})}.$$

#### 4.2.3 Antimycobacterial Activity

The antimycobacterial activity was evaluated using the broth microdilution method. In flat‐bottom microtitration plates, MIC was determined using Middlebrook 7H9 Broth Base (SEVAC, Prague, Czech Republic). The final volume in each well was 200 µL, consisting of 100 µL of the tested compound solution and 100 µL of the mycobacterial inoculum. The inoculum was prepared to achieve a cell density of 3 × 10⁵ Colony Forming Units/mL (CFU/mL). Thus, the final bacterial load was 1.5 × 10⁵ CFU/mL per well. MIC values were determined visually. Compounds were dissolved in DMSO and INH in sterile distilled water and added to the medium, resulting in a final 1% DMSO (^v^/_v_) concentration that did not affect mycobacterial growth. The mycobacterial strains included drug‐sensitive *M. tuberculosis* strain CNCTC 331/88 (Czech National Collection of Type Microorganisms; H_37_Rv) and two non‐tuberculous species: *M. avium* ssp. *avium* CNCTC 330/88 [resistant to INH, rifampicin (RIF), rifabutin, ofloxacin (OFX), and ethambutol (EMB)], and a clinical isolate of *M. kansasii* (6509/96). MIC values were determined using a two‐fold serial dilution method ranging from 1000 to 1 μM. MIC (in μM) represented the lowest concentration inhibiting completely mycobacterial growth after 14 and 21 days of incubation at 37°C, with an additional 7‐day assessment for *M. kansasii*. INH, a first‐line antitubercular drug, was used as a reference drug for MIC comparison.

#### 4.2.4 Antibacterial Activity

The antibacterial activity was evaluated against four Gram‐positive and four Gram‐negative bacterial strains of clinical importance: *S. aureus* ATCC (American Type Culture Collection) 29213, CCM (Czech Collection of Microorganisms) 4223 (SA); methicillin‐resistant *S. aureus* (MRSA) ATCC 43300, CCM 4750; *S. epidermidis* ATCC 12228, CCM 4418 (SE); *E. faecalis* ATCC 29212, CCM 4224 (EF); *E. coli* ATCC 25922, CCM 3954 (EC); *Klebsiella pneumoniae* ATCC 10031, CCM 4415; *Acinetobacter baumannii* ATCC 19606, DSM 30007; and *Pseudomonas aeruginosa* ATCC 27853, CCM 3955. The bacterial strains were obtained from the Czech Collection of Microorganisms (CCM, Brno, Czech Republic) or the German Collection of Microorganisms and Cell Cultures GmbH (DSM, Braunschweig, Germany).

This activity was determined using the broth microdilution method as per EUCAST (The European Committee on Antimicrobial Susceptibility Testing) recommendations [53], with slight modifications, in 96‐well plates. The final volume of the cultivation medium containing the tested compound(s) at concentrations ranging from 0.98 µM to 500 µM, along with the bacterial suspensions, was adjusted to 210 µL. This same volume was maintained for both positive and negative controls. The inoculum was prepared using Cation‐Adjusted Mueller–Hinton broth (CA‐MHB, M‐H 2 Broth). Bacterial suspensions were made from 16 to 24‐h‐old bacterial cultures and their turbidity was measured. The inoculum was then adjusted with CA‐MHB to reach a final cell density of approximately 1 × 10⁶ CFU/mL. A volume of 10 µL of bacterial suspension was added to each well, except in the negative controls, where 10 µL of CA‐MHB was used instead. The final bacterial load per well (except in negative controls) was approximately 5 × 10⁵ CFU/mL (range: 3–7 × 10⁵ CFU/mL), as recommended by EUCAST guidelines. This cell density corresponds to approximately 6 × 10⁴–1.5 × 10⁵ CFU per well. Compounds were dissolved in DMSO to prepare stock solutions. The final concentration of DMSO in the testing medium did not exceed 1% (^v^/_v_) and did not impact bacterial growth. All chemicals were purchased from Merck KGaA (Darmstadt, Germany). Antibacterial activity was quantified as MIC (reported in µM) after 24 and 48 h of static incubation in a dark, humidified atmosphere at 35 ± 2°C. MIC was determined visually by the naked eye in the well containing the lowest drug concentration, where no visible microbial growth was registered. Positive controls consisted of microbes alone, while negative controls comprised medium with DMSO. Standard antibiotics (piperacillin, ciprofloxacin, and gentamicin – data not shown) and internal quality controls were included in antibacterial activity testing.

#### 4.2.5 Antifungal Activity

Antifungal activity was evaluated against two strains: a yeast *Candida albicans* ATCC 24443, CCM 8320 (CA) and a filamentous fungus (mould) *Trichophyton interdigitale* (TI) ATCC 9533, CCM 8377. The broth microdilution method was performed following EUCAST guidelines [54, 55], with minor modifications.

The antifungal activity screening was conducted using 96‐well microplates. The final volume of the cultivation medium, containing the tested compound(s) at a concentration range of 0.98 µM to 500 µM and fungal suspensions, was 210 µL per well. The same final volume was maintained for both the positive and negative controls. RPMI‐1640 medium, supplemented with 2% glucose (^w^/_v_) and buffered to pH 7.0 using 3‐(*N*‐morpholino)propane‐1‐sulphonic acid, was used to prepare the yeast inoculum. An 18–24‐h‐old yeast culture was adjusted based on turbidity measurements and further diluted with the above‐mentioned RPMI‐1640 medium to achieve a final cell density of approximately 1–5 × 10⁶ CFU/mL. A 10 µL of the prepared yeast suspension was added to each well, except for the negative controls, where 10 µL of RPMI‐1640 medium was used. The final yeast cell density per well (except for negative controls) was approximately 1–5 × 10⁵ CFU/mL, consistent with the EUCAST recommendations. This corresponds to approximately 2 × 10⁴ to 1 × 10⁵ CFU per well. Compounds under investigation were dissolved in DMSO to prepare stock solutions. All media and chemicals were purchased from Merck KGaA (Darmstadt, Germany). The final concentration of DMSO in the test medium did not exceed 1% (^v^/_v_), ensuring it did not inhibit fungal growth. Incubation was performed statically in a dark, humidified atmosphere at 35 ± 2°C for 24 and 48 h for CA and 72 and 120 h for TI. MIC endpoints were determined visually, identifying the lowest drug concentration in wells where no visible microbial growth occurred. Positive controls comprised the fungus alone, while negative controls were medium with DMSO. Two commonly used standard antifungal drugs (amphotericin B and voriconazole; data not shown) were included as reference standards for testing antifungal activity against selected quality control yeast strains.

#### 4.2.6 Cytostatic Assays

For the cytostatic assays, A2058 (melanoma, derived from metastatic site, lymph node), Calu‐1 (epidermoid, bronchial carcinoma, non‐small cell lung cancer), HepG2 (hepatocellular carcinoma), MonoMac‐6 (monocytic leukaemia), HT‐29 (human colorectal carcinoma), Caco‐2 (human colon adenocarcinoma) and Vero E6 (a.k.a. VERO C1008) non‐tumorous kidney cells from an African green monkey (*C. sabaeus*) cell cultures were used.

Cell culturing. Calu‐1 and Caco‐2 was purchased from Sigma, and MonoMac‐6 was from the Deutsche Sammlung von Mikroorganismen und Zellkulturen, DSMZ (no.: ACC 124). VERO E6 cells were obtained from the European Collection of Authenticated Cell Cultures (ECACC 85020206) and was a generous gift from Dr. Zoltán Kis and Bernadett Pályi, National Center for Public Health and Pharmacy (NCPHP). All the other cell lines were generous gifts from Dr. József Tóvári (Department of Experimental Pharmacology, National Institute of Oncology, Budapest, Hungary).

For maintaining Caco‐2 cell culture DMEM supplemented with 10% FBS, 2 mM l‐glutamine, 100 µg/mL penicillin/streptomycin, 1 mM pyruvate, and 1% non‐essential amino acids (CM DMEM) was used. Vero E6 and Calu‐1 cells were maintained in DMEM high‐glucose (4.5 g/L) medium (Lonza) containing 10% FBS (Gibco) and supplemented with 2 mM of l‐glutamine (Lonza), 1 mM sodium pyruvate (Merck), and CellCultureGuard (PanReacApplichem). A2058, HepG2, HT‐29 and MonoMac‐6 was maintained in RPMI‐1640 medium (Lonza) containing 10% FBS supplemented with 2 mM l‐glutamine and 100 µg/mL penicillin/streptomycin (160 µg/mL gentamicin for MonoMac‐6).

Cells were cultured in sterile T25 and T75 flasks with a ventilation cap (Sarstedt, Nümbrecht, Germany) at 37°C in a humidified atmosphere with 5% CO_2_ in the ESCO Cell Culture Incubator (ESCO, Friedberg, Germany). Manipulations with the cells were performed in biosafety cabinet (laminar) ESCO Sentinel Gold class II model AC2‐4E8 (ESCO). No mycoplasma contamination was detected in the cell cultures.

Alamar Blue assay [56]. For the evaluation of the in vitro cytostatic activity of the compounds, the cell viability was determined on all tumorous and non‐tumorous cell cultures. After trypsinization and harvesting, 5 × 10^3^ cells per well were seeded in 96‐well plates with flat bottoms (Sarstedt) in 100 μL of serum‐containing (10% FBS) DMEM or RPMI growth medium (see the section above) and incubated at 37°C. After 24 h, cells were treated with various concentrations of the compounds (0.16 µM–100 µM) dissolved in serum‐free DMEM or RPMI medium, in volume of 100 µL (200 µL final volume in the well), and incubated for 24 h under standard conditions. The control wells were treated only with serum‐free medium. After 24 h of treatment, the cells were washed and cultured for additional 72 h. After than is incubation period, cells were washed twice with serum free medium and following that, they were cultured for another 72 h in 10% serum containing medium at 37°C. After that, cell viability vas determined by Alamar Blue assay. Alamar Blue is a non‐toxic, resazurin‐based dye that is reduced by living cells to a fluorescent molecule. Resazurin sodium salt (Merck, Darmstadt, Germany) was dissolved in PBS at *c* ═ 0.15 mg/mL, pH 7.4). 32.5 µL of the dye was added to each well and incubated at 37°C for 3–4 h (depends on the cell type) until the pink colour of the reduced dye appeared. Fluorescence intensity in each well was measured using a Synergy H4 multimode microplate reader (BioTek, Winooski, VT); at λex ═ 530/30 and λem ═ 610/10 nm. Cytostatic effect was calculated with the following equation:

Cytostaticeffect(%)═[1−(Fluorescenceintensity/treated/Fluorescenceintensity/control)]×100.

Cytostasis values were expressed as a percentage of the untreated control. 50 percent inhibitory concentration (IC_50_) was determined by fitting a sigmoid curve on the data points using Microcal™ Origin2021 software and the calculating X values at Y ═ 50 and expressed in micromolar units.

### 4.3 Molecular Docking

Human AChE and BChE crystallographic structures were obtained from a protein data bank (www.rcsb.org; pdb codes 4PQE and 1POI). The 3D structures of the ligands were prepared in Chem3D Pro 19.1 (ChemBioOffice 2019 Package, CambridgeSoft, Cambridge, MA, USA). In the preparation process, all water molecules were removed from the enzymes; the structures of enzymes and ligands were optimised using the UCSF Chimera software package [57] (Amber force field) and Autodock Tools 1.5.6 (ADT). Docking was performed using Autodock Vina [58] (a Lamarckian genetic algorithm was used). The 3D affinity grid box was designed to include all the active and peripheral sites of AChE and BChE. The number of grid points in the x‐, y‐ and z‐axes was 20, 20, and 20, with grid points separated by 1 Å (Autodock Vina). The most important amino acid residues (in AChE: Trp86, Trp286, Tyr72, Tyr124, Tyr133, Tyr337, and Tyr341) were set up as flexible. Graphic visualisations of the ligand‐enzyme interactions were prepared using PyMOL software (The PyMOL Molecular Graphics System, Version 1.5 Schrödinger LLC, New York, USA).

## Conflicts of Interest

The authors declare no conflicts of interest.

## Supporting information

ArchPharm_SupplMat_InChI_2020.

ESI_Revize.
