# Supplementary material for: Activity Profiling of Nitro‐Substituted Di(Hetero)Aryl 1,3,4‐ and 1,2,4‐Oxadiazoles: Antimicrobial, Cholinesterase Inhibition and Antioxidant Potential
Source: Arch Pharm (Weinheim). 2026 Jan 20;359(1):e70188. doi: 10.1002/ardp.70188 (PMC12820407; doi:10.1002/ardp.70188)
Supplement: Supplementary file 1 — ArchPharm_SupplMat_InChI_2020. [file ARDP-359-e70188-s001.doc]

**Supplemental Material: Novel Compounds and Biological Screening Results**

Activity Profiling of Nitro-Substituted Di(hetero)aryl 1,3,4- and 1,2,4-Oxadiazoles: Antimicrobial, Cholinesterase Inhibition and Antioxidant Potential

Enikő Šikorová^1^, Šárka Štěpánková^2^, Eva Frýbová^1^, Klára Konečná^3^, Jana Korbielová^4^, Markéta Švarcová^1^, Ondřej Janďourek^3^, Václav Pflégr^1^, Szilvia Bősze^5,6^, Martin Krátký^1^,*

1 Department of Organic and Bioorganic Chemistry, Faculty of Pharmacy in Hradec Králové, Charles University, Akademika Heyrovského 1203, 500 03 Hradec Králové, Czech Republic

2 Department of Biological and Biochemical Sciences, Faculty of Chemical Technology, University of Pardubice, Studentská 573, 532 10 Pardubice, Czech Republic

3 Department of Biological and Medical Sciences, Faculty of Pharmacy in Hradec Králové, Charles University, Akademika Heyrovského 1203, 500 03 Hradec Králové, Czech Republic

4 Laboratory for Mycobacterial Diagnostics and Tuberculosis, Regional Institute of Public Health in Ostrava, Partyzánské náměstí 7, Ostrava, Czech Republic

5 HUN-REN–ELTE Research Group of Peptide Chemistry, Hungarian Research Network, Institute of Chemistry, ELTE Eötvös Loránd University, Pázmány Péter sétány 1/A, H-1117 Budapest, Hungary

6 Department of Genetics, Cell- and Immunobiology, Faculty of Medicine, Semmelweis University, Nagyvárad tér 4, H-1089 Budapest, Hungary

Dr. Martin Krátký, Department of Organic and Bioorganic Chemistry, Faculty of Pharmacy in Hradec Králové, Charles University, Akademika Heyrovského 1203, 500 03, Hradec Králové, Czech Republic

| **Compound No.** | **InChI** | **Enzyme Inhibition (IC_50_; μM)^a^** | | **Antioxidant Capacity (TAC)^b^** | **Antimycobacterial Activity (μM)^c^** | **Antibacterial Activity (μM)^d^** | **Antifungal Activity (μM)^e^** | **Cytotoxicity (IC_50_; μM)^f^** |
| --- | --- | --- | --- | --- | --- | --- | --- | --- |
|  |  | **AChE** | **BuChE** |  |  |  |  |  |
| **1a** | InChI=1S/C13H10N4O4/c18-12(9-3-5-11(6-4-9)17(20)21)15-16-13(19)10-2-1-7-14-8-10/h1-8H,(H,15,18)(H,16,19) | 50.16±1.13 | 170.27±6.82 | - | - | - | - | - |
| **1b** | InChI=1S/C13H10N4O4/c18-12(9-3-1-5-11(7-9)17(20)21)15-16-13(19)10-4-2-6-14-8-10/h1-8H,(H,15,18)(H,16,19) | 41.18±1.56 | 200.67±3.07 | - | - | - | - | - |
| **1c** | InChI=1S/C13H10N4O4/c18-12(9-4-3-7-14-8-9)15-16-13(19)10-5-1-2-6-11(10)17(20)21/h1-8H,(H,15,18)(H,16,19) | 69.41±3.96 | 179.45±6.63 | - | - | - | - | - |
| **1d** | InChI=1S/C13H9N5O6/c19-12(8-2-1-5-14-7-8)15-16-13(20)10-4-3-9(17(21)22)6-11(10)18(23)24/h1-7H,(H,15,19)(H,16,20) | 53.40±1.83 | 380.63±7.08 | - | - | - | - | >100 |
| **1e** | InChI=1S/C13H9N5O6/c19-12(8-2-1-3-14-7-8)15-16-13(20)9-4-10(17(21)22)6-11(5-9)18(23)24/h1-7H,(H,15,19)(H,16,20) | 51.29±0.45 | 347.65±35.13 | - | 125 (*Mtb.*) | - | - | >100 |
| **1f** | InChI=1S/C17H12N4O4/c22-16(11-6-8-12(9-7-11)21(24)25)19-20-17(23)14-3-1-5-15-13(14)4-2-10-18-15/h1-10H,(H,19,22)(H,20,23) | 62.67±2.27 | 288.91±5.29 | - | - | - | - | - |
| **1g** | InChI=1S/C17H12N4O4/c22-16(11-4-1-5-12(10-11)21(24)25)19-20-17(23)14-6-2-8-15-13(14)7-3-9-18-15/h1-10H,(H,19,22)(H,20,23) | 58.88±4.09 | 230.90±4.40 | - | - | - | - | - |
| **1h** | InChI=1S/C17H12N4O4/c22-16(12-6-3-8-14-11(12)7-4-10-18-14)19-20-17(23)13-5-1-2-9-15(13)21(24)25/h1-10H,(H,19,22)(H,20,23) | 50.01±1.02 | 197.08±7.78 | - | - | - | - | - |
| **1i** | InChI=1S/C17H11N5O6/c23-16(12-3-1-5-14-11(12)4-2-8-18-14)19-20-17(24)13-7-6-10(21(25)26)9-15(13)22(27)28/h1-9H,(H,19,23)(H,20,24) | 92.00±5.26 | 398.44±8.48 | - | ≥1000 (*Mtb.*)  500 (*M. kansasii*) | - | - | - |
| **-1j** | InChI=1S/C17H11N5O6/c23-16(10-7-11(21(25)26)9-12(8-10)22(27)28)19-20-17(24)14-3-1-5-15-13(14)4-2-6-18-15/h1-9H,(H,19,23)(H,20,24) | 37.67±1.89 | 232.59±3.65 | - | - | - | - | - |
| **2a** | InChI=1S/C13H8N4O3/c18-17(19)11-5-3-9(4-6-11)12-15-16-13(20-12)10-2-1-7-14-8-10/h1-8H | 12.13±0.23 | **85.86±1.83** | 22.5 (12.13 μM)  69.0 (121.3 μM) | - | - | - | - |
| **2b** | InChI=1S/C13H8N4O3/c18-17(19)11-5-1-3-9(7-11)12-15-16-13(20-12)10-4-2-6-14-8-10/h1-8H | **6.68±0.12** | 101.68±2.06 | 21.0 (6.7 μM)  22.5 (67 μM) | ≥250 (*Mtb.*) | - | - | - |
| **2c** | InChI=1S/C13H8N4O3/c18-17(19)11-6-2-1-5-10(11)13-16-15-12(20-13)9-4-3-7-14-8-9/h1-8H | 19.46±0.12 | 137.73±6.47 | - | 1000 (*Mtb.*)  500-1000 (*M. kansasii*) | - | - | - |
| **2d** | InChI=1S/C13H7N5O5/c19-17(20)9-3-4-10(11(6-9)18(21)22)13-16-15-12(23-13)8-2-1-5-14-7-8/h1-7H | **7.15±0.40** | 322.32±5.55 | 20.5 (7.15 μM)  20.0 (71.5 μM) | 2 (*Mtb.*) | 125-250 (*S. aureus*)  15.62-31.25 (MRSA)  7.81-15.62 (*S. epidermidis*)  250 (*E. faecalis*) | 125 (*C. albicans*, *T. intedigitale*) | >100 |
| **2e** | InChI=1S/C13H7N5O5/c19-17(20)10-4-9(5-11(6-10)18(21)22)13-16-15-12(23-13)8-2-1-3-14-7-8/h1-7H | **1.47±0.05** | 140.82±6.21 | - | 2 (*Mtb.*)  32 (*M. avium*)  4-8 (*M. kansasii*) | 250 (*S. aureus*, MRSA, *E. faecalis*)  31.25-62.5 (*S. epidermidis*) | - | ≥100 (Calu-1, HT-29, Caco-2, Vero)  76.2 (HepG2)  89.2 (A2058)  78.2 (MonoMac-6) |
| **2f** | InChI=1S/C17H10N4O3/c22-21(23)12-8-6-11(7-9-12)16-19-20-17(24-16)14-3-1-5-15-13(14)4-2-10-18-15/h1-10H | 59.88±3.93 | 233.29±4.93 | - | - | - | - | - |
| **2g** | InChI=1S/C17H10N4O3/c22-21(23)12-5-1-4-11(10-12)16-19-20-17(24-16)14-6-2-8-15-13(14)7-3-9-18-15/h1-10H | 20.66±0.73 | 174.65±9.09 | - | - | - | - | - |
| **2h** | InChI=1S/C17H10N4O3/c22-21(23)15-9-2-1-5-13(15)17-20-19-16(24-17)12-6-3-8-14-11(12)7-4-10-18-14/h1-10H | 30.36±1.30 | 187.81±1.37 | - | - | - | - | - |
| **2i** | InChI=1S/C17H9N5O5/c23-21(24)10-6-7-13(15(9-10)22(25)26)17-20-19-16(27-17)12-3-1-5-14-11(12)4-2-8-18-14/h1-9H | 29.21±0.04 | 346.08±6.94 | - | 4-8 (*Mtb.*) | - | - | - |
| **2j** | InChI=1S/C17H9N5O5/c23-21(24)11-7-10(8-12(9-11)22(25)26)16-19-20-17(27-16)14-3-1-5-15-13(14)4-2-6-18-15/h1-9H | **2.64±0.02** | >500 | - | 4-8 (*Mtb.*) | - | - | >100 |
| **3a** | InChI=1S/C7H6N4O5/c8-7(9-12)4-1-5(10(13)14)3-6(2-4)11(15)16/h1-3,12H,(H2,8,9) | 26.37±0.09 | 65.05±3.42 | - | - | - | - | - |
| **3b** | InChI=1S/C10H9N3O/c11-10(13-14)8-3-1-5-9-7(8)4-2-6-12-9/h1-6,14H,(H2,11,13) | 63.26±2.82 | 60.93±3.25 | - | - | - | - | - |
| **4a** | InChI=1S/C13H7N5O5/c19-17(20)10-4-9(5-11(6-10)18(21)22)12-15-13(23-16-12)8-2-1-3-14-7-8/h1-7H | 22.07±1.09 | 150.75±3.91 | - | - | - | - | - |
| **4b** | InChI=1S/C13H7N5O5/c19-17(20)10-4-9(5-11(6-10)18(21)22)13-15-12(16-23-13)8-2-1-3-14-7-8/h1-7H | 18.26±0.09 | 45.09±0.44 | 25.0 (18.3 μM)  64.0 (183 μM) | - | - | - | >100 |
| **4c** | InChI=1S/C17H9N5O5/c23-21(24)11-7-10(8-12(9-11)22(25)26)16-19-17(27-20-16)14-3-1-5-15-13(14)4-2-6-18-15/h1-9H | 15.46±0.24 | 223.12±11.75 | - | - | - | - | - |
| **4d** | InChI=1S/C17H9N5O5/c23-21(24)11-7-10(8-12(9-11)22(25)26)17-19-16(20-27-17)14-3-1-5-15-13(14)4-2-6-18-15/h1-9H | 33.72±2.00 | 123.03±9.89 | - | - | - | - | - |

^a^ Inhibition of acetylcholinesterase and butyrylcholinesterase

The inhibitory activities for AChE and BChE were quantified by determining IC50 values using a spectrophotometric assay based on a modified Ellman's method. The final reaction volume was 2000 µL, with enzyme activity of 0.2 U/mL, substrate concentration of 40 µM for either acetylthiocholine or butyrylthiocholine, and 100 µM of 5,5′-dithiobis-(2-nitrobenzoic acid) for all reactions. The tested compounds were dissolved in DMSO and subsequently diluted with demineralized water to a concentration of 1000 µM. Each compound was tested at five different concentrations in the final reaction mixture. The final concentration of DMSO in all assays was maintained at 0.2%. All experiments were performed in triplicate. The average values of reaction rates (v₀ for the uninhibited reaction and vᵢ for the inhibited reaction) were used to plot the ratio v₀/vᵢ as a function of inhibitor concentration. IC_50_ values were calculated from corresponding regression equations, where y = 2 (based on the IC_50_ definition). Acetylcholinesterase from electric eel (*Electrophorus electricus*; EeAChE) and butyrylcholinesterase from equine serum (EqBChE) were utilized in the study.

^b^ Antioxidant activity determination

Total antioxidant capacity (TAC) was determined using a commercially available kit, the Antioxidant Assay Kit (Merck, Prague, Czech Republic). The kit has a linear detection range from 1.5 to 1000 µM Trolox equivalents. Trolox standards and reaction mixtures were prepared according to the instructions for the Antioxidant Assay Kit. The concentrations of investigated derivatives were chosen based on the determined IC_50_ values for AChE and BChE inhibition. A concentration equal to the IC_50_ value and 10 times higher for a specific derivative was always used. Investigated derivatives were diluted with ultrapure water. Trolox standards, studied derivatives, and blanks were always assayed in duplicate in separate wells of a transparent, flat bottom 96-well plate. Each well contained 20 µl of suitably diluted Trolox or tested derivative or ultrapure water in 100 µl of the reaction mixture. After 10 min incubation at room temperature, the absorbance was measured at 570 nm on BioTek Synergy H1 Multimode Reader. The dependence of absorbance A570 on the concentration of Trolox standard was plotted, and the slope of the standard curve was determined.

^c^ Antimycobacterial activity

The antimycobacterial activity was evaluated using the broth microdilution method. In flat-bottom microtitration plates, MIC was determined using Middlebrook 7H9 Broth Base. The final volume in each well was 200 µL, consisting of 100 µL of the tested compound solution and 100 µL of the mycobacterial inoculum. The inoculum was prepared to achieve a cell density of 3×10⁵ Colony Forming Units/mL (CFU/mL). Thus, the final bacterial load was 1.5×10⁵ CFU/mL per well. MIC values were determined visually. Compounds were dissolved in DMSO and added to the medium, resulting in a final 1% DMSO (v/v) concentration that did not affect mycobacterial growth. The mycobacterial strains included drug-sensitive *Mycobacterium tuberculosis* strain CNCTC 331/88 (Czech National Collection of Type Microorganisms; H_37_Rv) and two non-tuberculous species: *Mycobacterium avium* ssp. *avium* CNCTC 330/88 [resistant to isoniazid, rifampicin, rifabutin, ofloxacin, and ethambutol], and a clinical isolate of *Mycobacterium kansasii* (6509/96). MIC values were determined using a two-fold serial dilution method ranging from 1000 to 1 μM. MIC (in μM) represented the lowest concentration inhibiting completely mycobacterial growth after 14 and 21 days of incubation at 37°C, with an additional 7-day assessment for *M. kansasii*.

^d^ Antibacterial activity

The activity was evaluated against four Gram-positive and four Gram-negative bacterial strains of clinical importance: *Staphylococcus aureus* ATCC (American Type Culture Collection) 29213, CCM (Czech Collection of Microorganisms) 4223 (SA); methicillin-resistant *Staphylococcus aureus* (MRSA) ATCC 43300, CCM 4750; *Staphylococcus epidermidis* ATCC 12228, CCM 4418 (SE); *Enterococcus faecalis* ATCC 29212, CCM 4224 (EF); *Escherichia coli* ATCC 25922, CCM 3954 (EC); *Klebsiella pneumoniae* ATCC 10031, CCM 4415; *Acinetobacter baumannii* ATCC 19606, DSM 30007; and *Pseudomonas aeruginosa* ATCC 27853, CCM 3955. The bacterial strains were obtained from CCM (Brno, Czech Republic) or the German Collection of Microorganisms and Cell Cultures GmbH (DSM, Braunschweig, Germany). This activity was determined using the broth microdilution method as per EUCAST (The European Committee on Antimicrobial Susceptibility Testing) recommendations, with slight modifications, in 96-well plates. The final volume of the cultivation medium containing the tested compound(s) at concentrations ranging from 0.98 µM to 500 µM, along with the bacterial suspensions, was adjusted to 210 µL. This same volume was maintained for both positive and negative controls. The inoculum was prepared using Cation-Adjusted Mueller-Hinton broth (CA-MHB, M-H 2 Broth). Bacterial suspensions were made from 16-24 hour-old bacterial cultures and their turbidity was measured. The inoculum was then adjusted with CA-MHB to reach a final cell density of approximately 1×10⁶ CFU/mL. A volume of 10 µL of bacterial suspension was added to each well, except in the negative controls, where 10 µL of CA-MHB was used instead. The final bacterial load per well (except in negative controls) was approximately 5×10⁵ CFU/mL (range: 3–7×10⁵ CFU/mL), as recommended by EUCAST guidelines. This cell density corresponds to approximately 6×10⁴ – 1.5×10⁵ CFU per well. Compounds were dissolved in DMSO to prepare stock solutions. The final concentration of DMSO in the testing medium did not exceed 1% (v/v) and did not impact bacterial growth. Antibacterial activity was quantified as MIC (reported in µM) after 24 and 48 hours of static incubation in a dark, humidified atmosphere at 35±2 °C. MIC was determined visually by the naked eye in the well containing the lowest drug concentration, where no visible microbial growth was registered.

^e^ Antifungal activity

Antifungal activity was evaluated against two strains: a yeast *Candida albicans* ATCC 24443, CCM 8320 (CA) and a filamentous fungus (mould) *Trichophyton interdigitale* (TI) ATCC 9533, CCM 8377. The broth microdilution method was performed following EUCAST guidelines, with minor modifications. The antifungal activity screening was conducted using 96-well microplates. The final volume of the cultivation medium, containing the tested compound(s) at a concentration range of 0.98 µM to 500 µM and fungal suspensions, was 210 µL per well. The same final volume was maintained for both the positive and negative controls. RPMI-1640 medium, supplemented with 2% glucose (w/v) and buffered to pH 7.0 using 3-(*N*-morpholino)propane-1-sulphonic acid, was used to prepare the yeast inoculum. An 18–24-hour-old yeast culture was adjusted based on turbidity measurements and further diluted with the above-mentioned RPMI-1640 medium to achieve a final cell density of approximately 1–5×10⁶ CFU/mL. A 10 µL of the prepared yeast suspension was added to each well, except for the negative controls, where 10 µL of RPMI-1640 medium was used. The final yeast cell density per well (except for negative controls) was approximately 1–5×10⁵ CFU/mL, consistent with the EUCAST recommendations. This corresponds to approximately 2 × 10⁴ to 1 × 10⁵ CFU per well. Compounds under investigation were dissolved in DMSO to prepare stock solutions. The final concentration of DMSO in the test medium did not exceed 1% (v/v), ensuring it did not inhibit fungal growth. Incubation was performed statically in a dark, humidified atmosphere at 35±2 °C for 24 and 48 hours for CA and 72 and 120 hours for TI. MIC endpoints were determined visually, identifying the lowest drug concentration in wells where no visible microbial growth occurred.

^f^ Cytotoxicity evaluation

For the cytostatic assays, A2058 (melanoma), Calu-1 (epidermoid, bronchial carcinoma, non-small cell lung cancer), HepG2 (hepatocellular carcinoma), MonoMac-6 (monocytic leukemia), HT-29 (human colorectal carcinoma), Caco-2 (human colon adenocarcinoma) and Vero E6 non-tumorous kidney cells from an African green monkey cell cultures were used. After trypsinization and harvesting, 5×10^3^ cells per well were seeded in 96-well plates with flat bottoms in 100 μL of serum-containing (10% FBS) DMEM or RPMI growth medium and incubated at 37 °C. After 24 h, cells were treated with various concentrations of the compounds (0.16 µM – 100 µM) dissolved in serum-free DMEM or RPMI medium, in volume of 100 µL (200 µL final volume in the well), and incubated for 24 h under standard conditions. The control wells were treated only with serum-free medium. After 24 h of treatment, the cells were washed and cultured for additional 72 h. After than is incubation period, cells were washed twice with serum free medium and following that, they were cultured for another 72 hours in 10% serum containing medium at 37°C. After that, cell viability vas determined by Alamar Blue assay. Resazurin sodium salt was dissolved in PBS at c = 0.15 mg/mL, pH 7.4. 32.5 µL of the dye was added to each well and incubated at 37 °C for 3–4 h until the pink color of the reduced dye appeared. Fluorescence intensity in each well was measured using a Synergy H4 multimode microplate reader at λ_ex_ = 530/30 and λ_em_ = 610/10 nm. Cytostatic effect was calculated with the following equation: Cytostatic effect (%) = [1 ‒ (Fluorescence intensity/treated/Fluorescence intensity/control)]×100. Cytostasis values were expressed as a percentage of the untreated control. 50 percent inhibitory concentration (IC_50_) was determined by fitting a sigmoid curve on the data points using Microcal™ Origin2021 software and the calculating X values at Y=50 and expressed in micromolar units.
