# Supplementary material for: Activity Profiling of Nitro‐Substituted Di(Hetero)Aryl 1,3,4‐ and 1,2,4‐Oxadiazoles: Antimicrobial, Cholinesterase Inhibition and Antioxidant Potential
Source: Arch Pharm (Weinheim). 2026 Jan 20;359(1):e70188. doi: 10.1002/ardp.70188 (PMC12820407; doi:10.1002/ardp.70188)
Supplement: Supplementary file 2 — ESI_Revize. [file ARDP-359-e70188-s002.docx]

Supplementary material for

Activity Profiling of Nitro-Substituted Di(hetero)aryl 1,3,4- and 1,2,4-Oxadiazoles: Antimicrobial, Cholinesterase Inhibition and Antioxidant Potential

Enikő Šikorová^1^, Šárka Štěpánková^2^, Eva Frýbová^1^, Klára Konečná^3^, Jana Korbielová^4^, Markéta Švarcová^1^, Ondřej Janďourek^3^, Václav Pflégr^1^, Szilvia Bősze^5,6^, Martin Krátký^1,^*

^1^ Department of Organic and Bioorganic Chemistry, Faculty of Pharmacy in Hradec Králové, Charles University, Akademika Heyrovského 1203, 500 03 Hradec Králové, Czech Republic

^2^ Department of Biological and Biochemical Sciences, Faculty of Chemical Technology, University of Pardubice, Studentská 573, 532 10 Pardubice, Czech Republic

^3^ Department of Biological and Medical Sciences, Faculty of Pharmacy in Hradec Králové, Charles University, Akademika Heyrovského 1203, 500 03 Hradec Králové, Czech Republic

^4^ Laboratory for Mycobacterial Diagnostics and Tuberculosis, Regional Institute of Public Health in Ostrava, Partyzánské náměstí 7, Ostrava, Czech Republic

^5^ HUN-REN–ELTE Research Group of Peptide Chemistry, Hungarian Research Network, Institute of Chemistry, ELTE Eötvös Loránd University, Pázmány Péter sétány 1/A, H-1117 Budapest, Hungary

^6^ Department of Genetics, Cell- and Immunobiology, Faculty of Medicine, Semmelweis University, Nagyvárad tér 4, H-1089 Budapest, Hungary

*: Corresponding author: [martin.kratky@faf.cuni.cz](mailto:martin.kratky@faf.cuni.cz)

**Table of contents**

| Chapter | | Figures | Pages |
| --- | --- | --- | --- |
| 1. | **^1^H and ^13^C NMR spectra of compounds 1a-j, 2a-j, 3a-b, 4a-d** | **S1 – S52** | **S2-S53** |
| 2. | **The IC_50_ values graphs used for their calculations** | **S53 – S105** | **S54 – S71** |
| 3. | **Calibration of TAC equivalents** | **S106** | **S72** |

**1. ^1^H and ^13^C NMR spectra of compounds 1a-j, 2a-j, 3a-b, 4a-d**

**Figure S1** ^1^H NMR spectrum of *N*'-(4-nitrobenzoyl)nicotinohydrazide (**1a**)

**Figure S2** ^13^C NMR spectrum of *N*'-(4-nitrobenzoyl)nicotinohydrazide (**1a**)

**Figure S3** ^1^H NMR spectrum of *N'*-(3-nitrobenzoyl)nicotinohydrazide (**1b**)

**Figure S4** ^13^C NMR spectrum of *N'*-(3-nitrobenzoyl)nicotinohydrazide (**1b**)

**Figure S5** ^1^H NMR spectrum of *N'*-(2-nitrobenzoyl)nicotinohydrazide (**1c**)

**Figure S6** ^13^C NMR *N'*-(2-nitrobenzoyl)nicotinohydrazide (**1c**)

**Figure S7** ^1^H NMR spectrum of *N'*-(2,4-dinitrobenzoyl)nicotinohydrazide (**1d**)

**Figure S8** ^13^C NMR spectrum of *N'*-(2,4-dinitrobenzoyl)nicotinohydrazide (**1d**)

**Figure S9** ^1^H NMR spectrum of *N'*-(3,5-dinitrobenzoyl)nicotinohydrazide (**1e**)

**Figure S10** ^13^C NMR spectrum of *N'*-(3,5-dinitrobenzoyl)nicotinohydrazide (**1e**)

**Figure S11** ^1^H NMR spectrum of ***N'*-(4-nitrobenzoyl)quinoline-5-carbohydrazide (1f**)

**Figure S12** ^13^C NMR spectrum of ***N'*-(4-nitrobenzoyl)quinoline-5-carbohydrazide (1f**)

**Figure S13** ^1^H NMR spectrum of ***N'*-(3-nitrobenzoyl)quinoline-5-carbohydrazide (1g**)

**Figure S14** ^13^C NMR spectrum of ***N'*-(3-nitrobenzoyl)quinoline-5-carbohydrazide (1g**)

**Figure S15** ^1^H NMR spectrum of ***N'*-(2-nitrobenzoyl)quinoline-5-carbohydrazide (1h**)

**Figure S16** ^13^C NMR spectrum of ***N'*-(2-nitrobenzoyl)quinoline-5-carbohydrazide (1h**)

**Figure S17** ^1^H NMR spectrum of ***N'*-(2,4-dinitrobenzoyl)quinoline-5-carbohydrazide (1i**)

**Figure S18** ^13^C NMR spectrum of ***N'*-(2,4-dinitrobenzoyl)quinoline-5-carbohydrazide (1i**)

**Figure S19** ^1^H NMR spectrum of ***N'*-(3,5-dinitrobenzoyl)quinoline-5-carbohydrazide (1j**)

**Figure S20** ^13^C NMR spectrum of ***N'*-(3,5-dinitrobenzoyl)quinoline-5-carbohydrazide (1j**)

**Figure S21** ^1^H NMR spectrum of **2-(4-nitrophenyl)-5-(pyridin-3-yl)-1,3,4-oxadiazole** (**2a**)

**Figure S22** ^13^C NMR spectrum of **2-(4-nitrophenyl)-5-(pyridin-3-yl)-1,3,4-oxadiazole** (**2a**)

**Figure S23** ^1^H NMR spectrum of **2-(3-nitrophenyl)-5-(pyridin-3-yl)-1,3,4-oxadiazole** (**2b**)

**Figure S24** ^13^C NMR spectrum of **2-(3-nitrophenyl)-5-(pyridin-3-yl)-1,3,4-oxadiazole** (**2b**)

**Figure S25** ^1^H NMR spectrum of **2-(2-nitrophenyl)-5-(pyridin-3-yl)-1,3,4-oxadiazole** (**2c**)

**Figure S26** ^13^C NMR spectrum of **2-(2-nitrophenyl)-5-(pyridin-3-yl)-1,3,4-oxadiazole** (**2c**)

**Figure S27** ^1^H NMR spectrum of **2-(2,4-dinitrophenyl)-5-(pyridin-3-yl)-1,3,4-oxadiazole** (**2d**)

**Figure S28** ^13^C NMR spectrum of **2-(2,4-dinitrophenyl)-5-(pyridin-3-yl)-1,3,4-oxadiazole** (**2d**)

**Figure S29** ^1^H NMR spectrum of **2-(3,5-dinitrophenyl)-5-(pyridin-3-yl)-1,3,4-oxadiazole** (**2e**)

**Figure S30** ^13^C NMR spectrum of **2-(3,5-dinitrophenyl)-5-(pyridin-3-yl)-1,3,4-oxadiazole** (**2e**)

**Figure S31** ^1^H NMR spectrum of **2-(4-nitrophenyl)-5-(quinolin-5-yl)-1,3,4-oxadiazole (2f**)

**Figure S32** ^13^C NMR spectrum of **2-(4-nitrophenyl)-5-(quinolin-5-yl)-1,3,4-oxadiazole (2f**)

**Figure S33** ^1^H NMR spectrum of **2-(3-nitrophenyl)-5-(quinolin-5-yl)-1,3,4-oxadiazole (2g**)

**Figure S34** ^13^C NMR spectrum of **2-(3-nitrophenyl)-5-(quinolin-5-yl)-1,3,4-oxadiazole (2g**)

**Figure S35** ^1^H NMR spectrum of **2-(2-nitrophenyl)-5-(quinolin-5-yl)-1,3,4-oxadiazole (2h**)

**Figure S36** ^13^C NMR spectrum of **2-(2-nitrophenyl)-5-(quinolin-5-yl)-1,3,4-oxadiazole (2h**)

**Figure S37** ^1^H NMR spectrum of **2-(2,4-dinitrophenyl)-5-(quinolin-5-yl)-1,3,4-oxadiazole (2i**)

**Figure S38** ^13^C NMR spectrum of **2-(2,4-dinitrophenyl)-5-(quinolin-5-yl)-1,3,4-oxadiazole (2i**)

**Figure S39** ^1^H NMR spectrum of **2-(3,5-dinitrophenyl)-5-(quinolin-5-yl)-1,3,4-oxadiazole (2j**)

**Figure S40** ^13^C NMR spectrum of **2-(3,5-dinitrophenyl)-5-(quinolin-5-yl)-1,3,4-oxadiazole (2j**)

**Figure S41** ^1^H NMR spectrum of ***N*'-hydroxy-3,5-dinitrobenzimidamide** (**3a**)

**Figure S42** ^13^C NMR spectrum of ***N*'-hydroxy-3,5-dinitrobenzimidamide** (**3a**)

**Figure S43** ^1^H NMR spectrum of ***N*'-hydroxyquinoline-5-carboximidamide** (**3b**)

**Figure S44** ^13^C NMR spectrum of ***N*'-hydroxyquinoline-5-carboximidamide** (**3b**)

**Figure S45** ^1^H NMR spectrum of **3-(3,5-dinitrophenyl)-5-(pyridin-3-yl)-1,2,4-oxadiazole (4a**)

**Figure S46** ^13^C NMR spectrum of **3-(3,5-dinitrophenyl)-5-(pyridin-3-yl)-1,2,4-oxadiazole (4a**)

**Figure S47** ^1^H NMR spectrum of **5-(3,5-dinitrophenyl)-3-(pyridin-3-yl)-1,2,4-oxadiazole (4b**)

**Figure S48** ^13^C NMR spectrum of **5-(3,5-dinitrophenyl)-3-(pyridin-3-yl)-1,2,4-oxadiazole (4b**)

**Figure S49** ^1^H NMR spectrum of **3-(3,5-dinitrophenyl)-5-(quinolin-5-yl)-1,2,4-oxadiazole (4c**)

**Figure S50** ^13^C NMR spectrum of **3-(3,5-dinitrophenyl)-5-(quinolin-5-yl)-1,2,4-oxadiazole (4c**)

**Figure S51** ^1^H NMR spectrum of **5-(3,5-dinitrophenyl)-3-(quinolin-5-yl)-1,2,4-oxadiazole (4d**)

**Figure S52** ^13^C NMR spectrum of **5-(3,5-dinitrophenyl)-3-(quinolin-5-yl)-1,2,4-oxadiazole (4d**)

**2. The IC_50_ values graphs used for their calculations**

**Figure S53** The dependence v_0_/v_i_ vs. concentration of inhibitor **1a** inhibiting eeAChE

**Figure S54** The dependence v_0_/v_i_ vs. concentration of inhibitor **1a** inhibiting eqBChE

**Figure S55** The dependence v_0_/v_i_ vs. concentration of inhibitor **1b** inhibiting eeAChE

**Figure S56** The dependence v_0_/v_i_ vs. concentration of inhibitor **1b** inhibiting eqBChE

**Figure S57** The dependence v_0_/v_i_ vs. concentration of inhibitor **1c** inhibiting eeAChE

**Figure S58** The dependence v_0_/v_i_ vs. concentration of inhibitor **1c** inhibiting eqBChE

**Figure S59** The dependence v_0_/v_i_ vs. concentration of inhibitor **1d** inhibiting eeAChE

**Figure S60** The dependence v_0_/v_i_ vs. concentration of inhibitor **1d** inhibiting eqBChE

**Figure S61** The dependence v_0_/v_i_ vs. concentration of inhibitor **1e** inhibiting eeAChE

**Figure S62** The dependence v_0_/v_i_ vs. concentration of inhibitor **1e** inhibiting eqBChE

**Figure S63** The dependence v_0_/v_i_ vs. concentration of inhibitor **1f** inhibiting eeAChE

**Figure S64** The dependence v_0_/v_i_ vs. concentration of inhibitor **1f** inhibiting eqBChE

**Figure S65** The dependence v_0_/v_i_ vs. concentration of inhibitor **1g** inhibiting eeAChE

**Figure S66** The dependence v_0_/v_i_ vs. concentration of inhibitor **1g** inhibiting eqBChE

**Figure S67** The dependence v_0_/v_i_ vs. concentration of inhibitor **1h** inhibiting eeAChE

**Figure S68** The dependence v_0_/v_i_ vs. concentration of inhibitor **1h** inhibiting eqBChE

**Figure S69** The dependence v_0_/v_i_ vs. concentration of inhibitor **1i** inhibiting eeAChE

**Figure S70** The dependence v_0_/v_i_ vs. concentration of inhibitor **1i** inhibiting eqBChE

**Figure S71** The dependence v_0_/v_i_ vs. concentration of inhibitor **1j** inhibiting eeAChE

**Figure S72** The dependence v_0_/v_i_ vs. concentration of inhibitor **1j** inhibiting eqBChE

**Figure S73** The dependence v_0_/v_i_ vs. concentration of inhibitor **2a** inhibiting eeAChE

**Figure S74** The dependence v_0_/v_i_ vs. concentration of inhibitor **2a** inhibiting eqBChE

**Figure S75** The dependence v_0_/v_i_ vs. concentration of inhibitor **2b** inhibiting eeAChE

**Figure S76** The dependence v_0_/v_i_ vs. concentration of inhibitor **2b** inhibiting eqBChE

**Figure S77** The dependence v_0_/v_i_ vs. concentration of inhibitor **2c** inhibiting eeAChE

**Figure S78** The dependence v_0_/v_i_ vs. concentration of inhibitor **2c** inhibiting eqBChE

**Figure S79** The dependence v_0_/v_i_ vs. concentration of inhibitor **2d** inhibiting eeAChE

**Figure S80** The dependence v_0_/v_i_ vs. concentration of inhibitor **2d** inhibiting eqBChE

**Figure S81** The dependence v_0_/v_i_ vs. concentration of inhibitor **2e** inhibiting eeAChE

**Figure S82** The dependence v_0_/v_i_ vs. concentration of inhibitor **2e** inhibiting eqBChE

**Figure S83** The dependence v_0_/v_i_ vs. concentration of inhibitor **2f** inhibiting eeAChE

**Figure S84** The dependence v_0_/v_i_ vs. concentration of inhibitor **2f** inhibiting eqBChE

**Figure S85** The dependence v_0_/v_i_ vs. concentration of inhibitor **2g** inhibiting eeAChE

**Figure S86** The dependence v_0_/v_i_ vs. concentration of inhibitor **2g** inhibiting eqBChE

**Figure S87** The dependence v_0_/v_i_ vs. concentration of inhibitor **2h** inhibiting eeAChE

**Figure S88** The dependence v_0_/v_i_ vs. concentration of inhibitor **2h** inhibiting eqBChE

**Figure S89** The dependence v_0_/v_i_ vs. concentration of inhibitor **2i** inhibiting eeAChE

**Figure S90** The dependence v_0_/v_i_ vs. concentration of inhibitor **2i** inhibiting eqBChE

**Figure S91** The dependence v_0_/v_i_ vs. concentration of inhibitor **2j** inhibiting eeAChE

Graph for **2j** inhibiting eqBChE is not provided (IC_50_ >500 µM).

**Figure S92** The dependence v_0_/v_i_ vs. concentration of inhibitor **3a** inhibiting eeAChE

**Figure S93** The dependence v_0_/v_i_ vs. concentration of inhibitor **3a** inhibiting eqBChE

**Figure S94** The dependence v_0_/v_i_ vs. concentration of inhibitor **3b** inhibiting eeAChE

**Figure S95** The dependence v_0_/v_i_ vs. concentration of inhibitor **3b** inhibiting eqBChE

**Figure S96** The dependence v_0_/v_i_ vs. concentration of inhibitor **4a** inhibiting eeAChE

**Figure S97** The dependence v_0_/v_i_ vs. concentration of inhibitor **4a** inhibiting eqBChE

**Figure S98** The dependence v_0_/v_i_ vs. concentration of inhibitor **4b** inhibiting eeAChE

**Figure S99** The dependence v_0_/v_i_ vs. concentration of inhibitor **4b** inhibiting eqBChE

**Figure S100** The dependence v_0_/v_i_ vs. concentration of inhibitor **4c** inhibiting eeAChE

**Figure S101** The dependence v_0_/v_i_ vs. concentration of inhibitor **4c** inhibiting eqBChE

**Figure S102** The dependence v_0_/v_i_ vs. concentration of inhibitor **4d** inhibiting eeAChE

**Figure S103** The dependence v_0_/v_i_ vs. concentration of inhibitor **4d** inhibiting eqBChE

**Figure S104** The dependence v_0_/v_i_ vs. concentration of rivastigmine inhibiting eeAChE

**Figure S105** The dependence v_0_/v_i_ vs. concentration of rivastigmine inhibiting eqBChE

**3. Calibration of Total Antioxidant Capacity (TAC) equivalents**

**Figure S106** The calibration of TAC
